# Dynamic changes of molecular pattern and cellular subpopulation in puncture-induced tendon injury model

**DOI:** 10.1016/j.isci.2025.112034

**Published:** 2025-02-19

**Authors:** Zizhan Huang, Ziyang Li, Dengfeng Ruan, Yiwen Xu, Honglu Cai, Hengzhi Liu, Haocheng Jin, Peiwen He, Yang Fei, Jiayun Huang, Canlong Wang, Xiao Chen, Jia Jiang, Weiliang Shen

**Affiliations:** 1 Department of Sports Medicine & Orthopedic Surgery, the Second Affiliated Hospital, Zhejiang University School of Medicine, Hangzhou City, Zhejiang Province, P.R. China; 2 Institute of Sports Medicine, Zhejiang University, Hangzhou City, Zhejiang Province, P.R. China; 3 Orthopedics Research Institute of Zhejiang University, Hangzhou City, Zhejiang Province, P.R. China; 4 Zhejiang Key Laboratory of Motor System Disease Precision Research and Therapy, Hangzhou City, Zhejiang Province, P.R. China; 5 Clinical Research Center of Motor System Disease of Hangzhou City, Hangzhou City, Zhejiang Province, P.R. China; 6 Department of Orthopedics, National Center for Orthopedics, Shanghai Sixth People’s Hospital Affiliated to Shanghai Jiao Tong University School of Medicine, Shanghai, P.R. China; 7 Dr. Li Dak Sum-Yip Yio Chin Center for Stem Cells and Regenerative Medicine, Zhejiang University School of Medicine, Hangzhou, P.R. China; 8 Liangzhu Laboratory, Zhejiang University Medical Center, 1369 West Wenyi Road, Hangzhou City, P.R. China; 9 State Key Laboratory of Transvascular Implantation Devices, Hangzhou City, Zhejiang Province, P.R. China; 10 China Orthopedic Regenerative Medicine Group (CORMed), Hangzhou, China

**Keywords:** Orthopedics, Cellular physiology

## Abstract

Tendon degeneration and injury often result in significant pain and functional impairment. Typically, tendon healing occurs through a scar-mediated response and may progress to chronic tendinopathy without timely intervention. However, the molecular mechanisms underlying early tendon repair remain poorly understood. Further investigation is also impeded by the limited availability of early tendon injury samples in clinical settings. In this study, we established a puncture-induced tendon injury model to investigate the molecular patterns and cellular subpopulations involved in early tendon injury across multiple time points. RNA sequencing identified seven gene sets with distinct expression profiles during the early stages of tendon injury. Single-cell RNA sequencing further revealed eight myeloid cell types and seven mesenchymal cell types participating in the tendon repair process. Together, these findings illuminate the molecular and cellular dynamics coordinating early tendon repair, providing insights that could inform future clinical treatments for tendinopathy and tendon injury.

## Highlights

• A stable puncture-induced tendon injury mouse model was established
• Early tendon repair peaks with proliferation/inflammatory genes, then shifts to differentiation/remodeling
• Myofibroblast and myeloid tenogenic cells drive early tendon repair, as shown by scRNA-seq

## Graphical abstract

**Figure:**
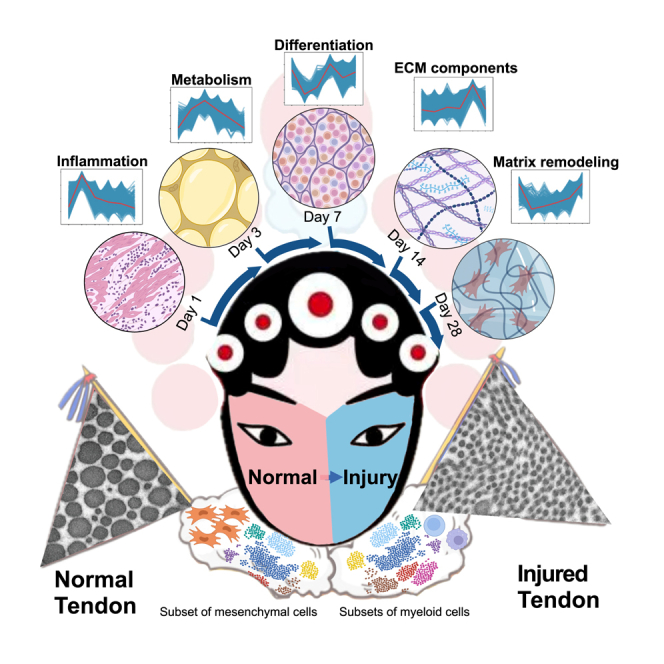

## Introduction

Tendon is an important structure connecting bone and muscle to achieve limb movement. With the aging of the population and the increase in the number of people participating in sports, the high incidence of tendon injuries, including chronic tendon injuries (tendinopathy) and acute tendon injuries (tendon rupture), seriously affect people’s quality of life and cause a huge social and economic burden.^1^ Currently, the treatments for tendon injuries include oral or local injection of drugs, physical therapy, and surgical suture. Although drugs can provide short-term analgesia, the long-term effect is not ideal. The results of studies on adjuvant therapy such as physical therapy are confusing and have not been widely promoted. These treatments usually only provide short-term relief of symptoms and have a high recurrence rate.^2^^,^^3^

The repair process after tendon injury can be divided into three distinct stages: inflammation, proliferation, and remodeling. During this process, tendon tissue undergoes a series of complex molecular and cellular changes, including extracellular matrix, cytokines, growth factors, and immune cells. However, our current understanding of the mechanisms in molecular and cellular changes during tendon repair after injury is still limited. Comprehensive molecular networks and cell lineage maps have not been established, and there is a lack of effective molecular and cellular markers to distinguish tendon tissues at different conditions after injury. These limitations have prevented us from developing a deep understanding in tendon repair mechanisms and innovative treatment strategies. Therefore, it is imperative to construct dynamic molecular networks and cell atlas.

Moreover, the limited availability of human specimens of early tendon injury hinder a thorough investigation of the repair mechanisms. Hence, it is necessary and valuable to use animal models to analyze the states of early tendon injury. In this study, we successfully established a robust early tendon microinjury model and elucidated the changes in transcriptional dynamics during tendon repair using high-throughput RNA-seq and scRNA-seq. RNA-seq analysis revealed different gene expression patterns at different time points, elucidating molecular features and functional differences at different stages (inflammation, proliferation, remodeling). ScRNA-seq analysis revealed the existence of different subtypes of mesenchymal cells that undergo different biological processes, including migration, proliferation, differentiation and transformation. Furthermore, myeloid cells involve in the immune response and could transform into tenogenic cell during early stages of tendon injury, highlighting their significant potential in regulating tendon repair.

## Results

### Construction of a puncture-induced tendon injury mouse model

**Figure 1:**
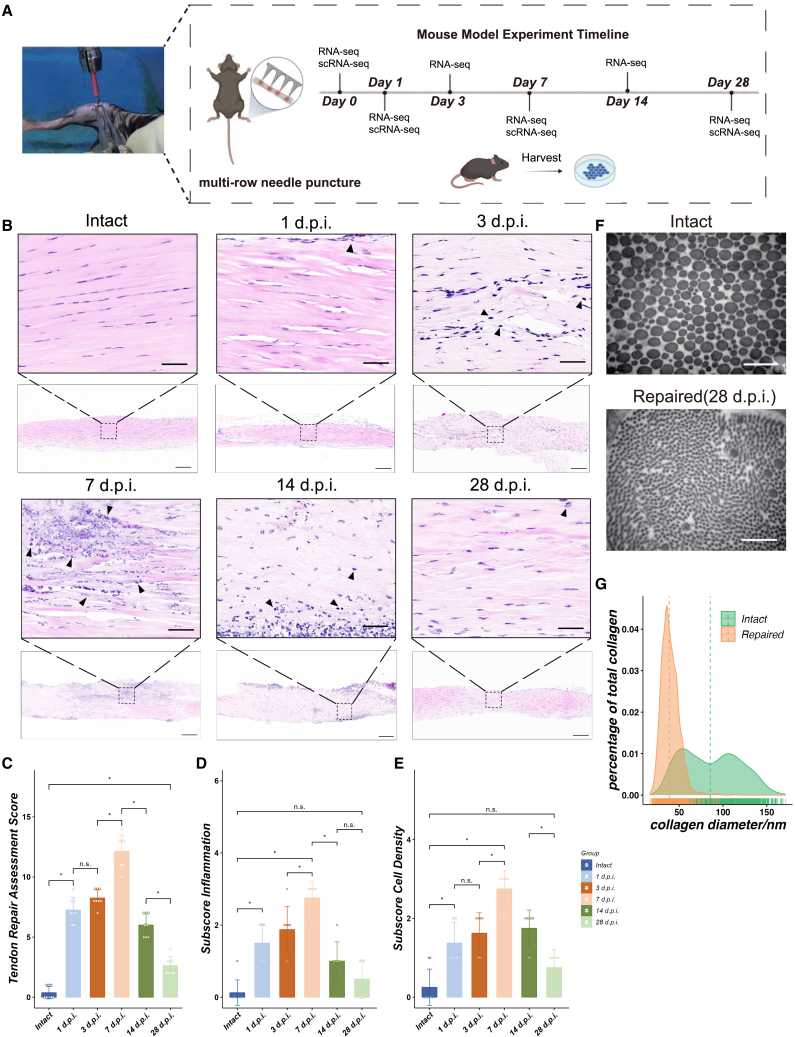
Establishment of the mouse model of tendon injury (A) Diagram depicting the experiment design adopted in this study. (B) Hematoxylin-eosin (HE) staining for different patellar tendon morphology after different time points post injury (l, 3, 7, 14, and 28 days). The box selected area were magnified and shown. The arrow indicates round inflammatory cells. Magnified area scale bar: 50μm. Low magnification view scale bar:200μm. (C–E) Calculation of scores based on the Movin’s tendon repair rating system and subscores for inflammatory infiltration area and cell density according to HE staining (*n* = 8). Mann-Whitney U test. Data are shown as mean ± SD. n.s. not significant, ∗*p* < 0.05. (F) Transmission electron microscope displaying microstructure of intact patellar tendon and the microstructure after 28 days post injury. Scale bar: 400nm. (G) The distribution of collagen diameters of intact or repaired patellar tendon.

Eight-week-old C57BL/6N mice underwent anesthesia and puncture-induced tendon injury (Figures 1A and S1A). The control group received only a surgical incision, followed by suturing the skin (referred as 0 days post injury, 0 d.p.i.). Tendon samples were collected at distinct intervals (0 d.p.i., 1 d.p.i., 3 d.p.i., 7 d.p.i., 14 d.p.i., 28 d.p.i.) for RNA-seq analysis and histological staining.

The histological results of HE staining of the tendon showed gradual changes in the organizational structure after injury. At 1 d.p.i. and 3 d.p.i., there was disruption and fragmentation of collagen fibers, resulting in a wavy and loose composition, together with infiltration of inflammatory cells on the surface of the tendon. The 7 d.p.i. group showed more pronounced infiltration, increased cell density and a larger area of inflammatory infiltration compared to the first two periods. However, the observed collagen disruption was significantly improved, while the fiber composition remained relatively loose and wavy. At 14 d.p.i., the arrangement of the collagen fibers gradually returned to normal, while inflammation was significantly reduced. However, cell density remained relatively high and some cells within the fibers lost their typical long fusiform morphology, resulting in increased cell roundness. Compared with intact group, 28 d.p.i. samples showed normalization of structure and collagen fiber arrangement, reduced inflammation, and reduced cell density that were not significantly different from intact tissue (Figure 1B).

Score using the semi-quantitative Movin rating system, covers six parameters: the fiber structure, fiber array, the nucleus roundness, the degree of inflammation, blood vessel growth and cell density. Each parameter is scored on a scale of 0–3, where 0 represents normal and 3 represents extremely abnormal (Table S1). The quantitative results of the assessment for tendon repair demonstrate that the total score of the 1 d.p.i. group significantly exceeded that of the intact group (*p* < 0.05). However, there were no significant differences between the 3 d.p.i. and 1 d.p.i. groups (*p* > 0.05). Moreover, the total score of the 7 d.p.i. group was higher than that of the 3 d.p.i. group (*p* < 0.05) and the 14 d.p.i. group (*p* < 0.05), while the total score of the 14 d.p.i. group exceeded that of the 28 d.p.i. group significantly (*p* < 0.05). Although visual perception showed no significant difference between the 28 d.p.i. group and the intact group, the quantitative results revealed that the former obtained significantly higher scores than the latter (*p* < 0.05) (Figures 1C–1E). In the 28 d.p.i. group, the gross morphological recovery of mouse tendons was similar to that in the 0 d.p.i. group, but the transmission electron microscopy showed that the collagen fibers changed from thick to thin (Figures 1F and 1G). Furthermore, micro-CT showed more tendon calcification in injured tendons after 6 weeks compared to the intact group (Figures S1B and S1C). These results indirectly demonstrate the incomplete repair after tendon injury.

### Analysis of differential gene expression post tendon injury

**Figure 2:**
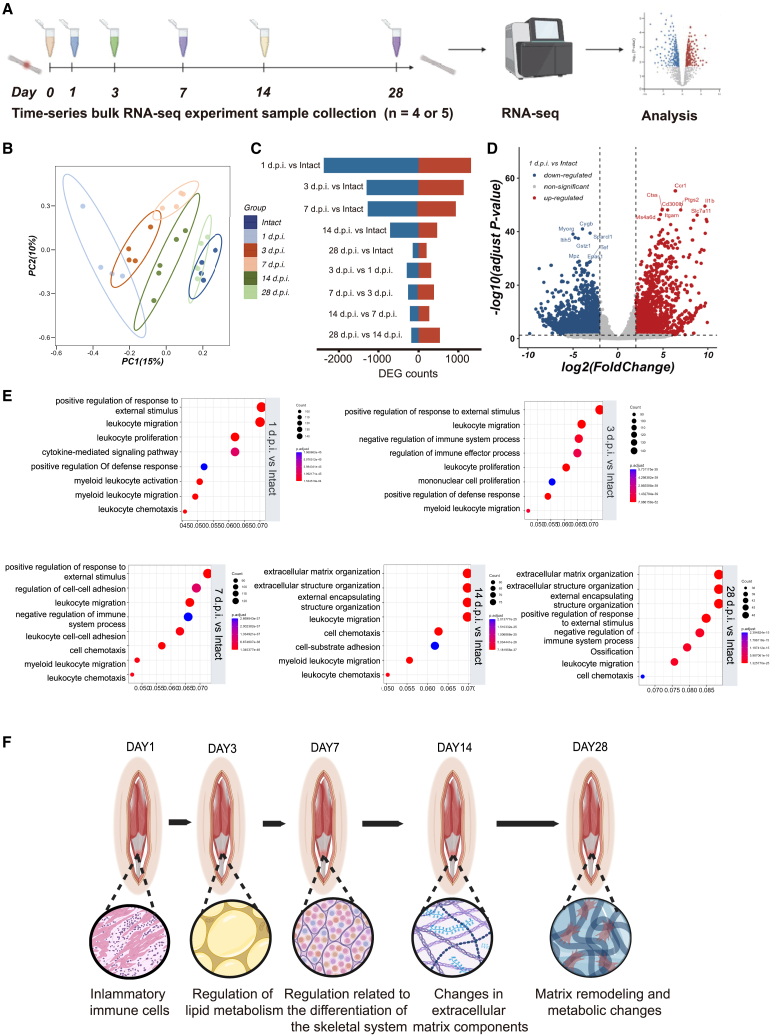
Differential analysis of gene expression after tendon injury at various time points (A) A graphical scheme of the patellar tendon sampling process of RNA-seq analysis. (B) Principal component analysis (PCA) results. (C) The number of differentially expressed genes between groups of adjacent time points and between the injured groups and the uninjured group (*n* = 4 or 5 per group). Upregulated genes are highlighted in red, indicating upregulated genes (log2FoldChange ≥1, adjusted *p* value < 0.05), and downregulated genes are presented in blue (log2FoldChange ≤ −1, adjusted *p* value < 0.05). (D) Volcano plot for differentially expressed genes between 1 d.p.i. and intact tendon (*n* = 4, |log2 FoldChange| ≥1, adjusted *p* value < 0.05). (E) Enrichment of upregulated genes in Gene Ontology (GO) biological process terms obtained by pairwise comparisons between the injured and uninjured groups (log2FoldChange ≥1, adjusted *p* value < 0.05). Top GO terms were determined based on the adjusted *p* value. (F) Summary plot showing typical changes of biological processes at different time points.

Subsequently, our aim was to specify the variations in gene expression during different stages of tendon healing in the tendon injury model. We performed bulk RNA-seq of tendon samples at different time points post injury (Figure 2A). After performing TPM normalization of raw counts, principal component analysis (PCA) was utilized to align each group within the same coordinate system. The biological replicates were well dispersed along the composition of PC1, and the intersection of confidence ellipses between the groups was minimized. These findings collectively confirm the reliability of the sequencing results and indicate minimal batch effects. Moreover, the 1 d.p.i. group exhibited the greatest deviation from the intact group on PC1, while the 3 d.p.i. group approached the intact group along PC1. The 7 d.p.i. and 14 d.p.i. groups were closer to the intact group. Lastly, the 28 d.p.i group was in the closet proximity to the intact group, as evidenced by the overlap of their confidence ellipses (Figure 2B).

DESeq2 was used to analyze transcriptome changes at each time point after injury. By conducting pairwise comparisons of differentially expressed genes (|log2FoldChange| ≥ 1 and adjusted *p*-value <0.05), it was observed that the 1 d.p.i. group had the most significant differential gene expressions among all pairwise comparisons, with 2,380 genes upregulated and 1,319 genes downregulated (Figures 2C and 2D). More genes were differentially expressed compared to the intact group at early time points after injury. At 28 d.p.i., 154 genes were upregulated and 189 genes were downregulated relative to the intact group (Figure 2C).

After performing differential gene analysis, we defined log2FoldChange ≥1 as significantly upregulated genes and log2FoldChange ≤ −1 as significantly downregulated genes. In the dot plot, we present the top GO terms with the smallest adjusted *p*-values. The color coding of the dot plot visually reflects the significance level of each term. We compared the injured tendon with the intact tendon and conducted GO enrichment analysis for both upregulated and downregulated genes. Our findings indicated that the genes upregulated in the 1 d.p.i. group were enriched in GO terms such as leukocyte migration, leukocyte chemotaxis, leukocyte activation, and positive response to external stimuli (Figure 2E).

Likewise, for 3 d.p.i. group, we observed upregulated gene enrichment in positive regulation of leukocyte migration, and leukocyte proliferation (Figure 2E). However, downregulated genes were enriched in GO terms related to regulation of lipid metabolism, lipid catabolic process and fatty acid metabolic process (Figure S2A). This may indicate a relative degree of inflammation and altered tissue metabolism.

Similarly, in the 7 d.p.i. group, upregulated genes were enriched in GO terms related to inflammation (Figure 2E). Downregulated genes were enriched in GO terms related to fatty acid metabolic process, muscle contraction, and muscle system process, implying regulation of fat metabolism and differentiation of the skeletal system at this stage (Figure S2A).

In contrast, for 14 d.p.i. group, the upregulated genes were enriched in GO terms related to the extracellular structure organization and extracellular matrix organization (Figure 2E). Inflammatory response-related GO terms such as leukocyte migration persisted, while downregulated genes were enriched in GO terms related to the response to various stimuli, implying a loss of function (Figure S2A). This suggests that repair process moved into the remodeling phase, and the extracellular matrix began to transform.

At 28 d.p.i., the number of differentially expressed genes was relatively small compared to the intact group. Upregulated genes were mainly enriched in GO terms related to the extracellular matrix organization, negative regulation of immune system process and ossification (Figure 2E). Downregulated genes were enriched in GO terms related to lipid catabolic process, fatty acid metabolic process and other metabolism-related processes (Figure S2A). This suggests that this stage is still in the period of matrix remodeling and metabolic changes.

Additionally, to minimize the potential confounding effect of age on experimental outcomes, we performed transcriptomic analysis on tendon tissues from both six-month-old and eight-week-old mice. The analysis revealed only 24 upregulated and 69 downregulated genes, demonstrating high transcriptomic similarity and no significant differences between the two age groups (Figures S2B and S2C). Finally, to illustrate the corresponding changes in biological processes at different time points during early tendon repair, we use pattern diagrams to summarize (Figure 2F).

### Analysis of temporal dynamic changes in gene expression post injury

**Figure 3:**
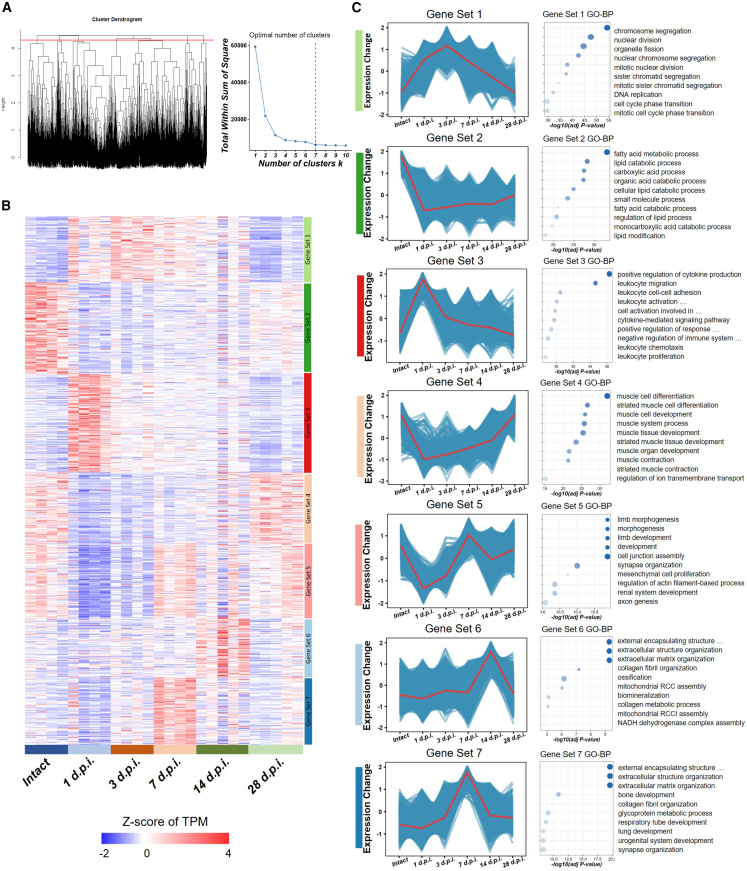
Mfuzz-based fuzzy clustering and enrichment analysis of DEGs in different clusters of genes (A) Combination of the unsupervised hierarchical cluster and the inflection point method were employed to determine the optimal number of clustering (*n* = 7). (B) Gene set-specific, sample-based differential gene expression heatmap. (C) Genes expression trends within each gene set were analyzed. Top GO terms were determined based on the adjusted *p* value.

Subsequently, we examined the patterns of gene expression during the initial phases of tendon injury. To begin with, we selected all differentially expressed genes across various groups at different time points, resulting in a total of 9,873 DEGs. Using the factoextra and fviz_nbclust package, we analyzed these genes and determined that the optimal number for clustering was seven (Figure 3A). Then, by analyzing the sample-based expression heatmap, we observed different gene expression characteristics at various time points during the tendon repair process (Figure 3B). Subsequently, we applied the Mfuzz algorithm to implement fuzzy clustering, dividing all differentially expressed genes into seven discrete gene sets, each displaying unique expression profiles (Figure 3C). The tendons of female mice show a similar transcriptomics trend compared to male mice, with seven clusters of functionally distinct gene sets identified (Figure S3).

Gene set 1, containing 1,235 genes, shows up-regulation peaking at 3 d.p.i., then down-regulation, primarily involving chromosome segregation, nuclear division, and cell cycle processes, indicating peak cell division and proliferation at 3 d.p.i. Gene set 2 includes 1,710 genes with decreased expression that gradually increases, enriched in lipid metabolism processes. Gene set 3, with 1,833 genes, shows up-regulation peaking at 1 d.p.i. and decrease afterward, linked to cytokine production and leukocyte activity, indicating an inflammatory response that resolves over time. Gene set 4 contains 1,324 genes with initial down-regulation followed by up-regulation from 1 d.p.i., related to muscle differentiation and development. Gene set 5, with 1,381 genes, has a wave-like pattern, peaking at 7 d.p.i., involved in limb morphogenesis and possibly tendon-resident stem cell activity during repair. Gene set 6 includes 1,126 genes with late-rising expression, peaking at 14 d.p.i., related to extracellular matrix remodeling and collagen processes. This indicates significant extracellular matrix remodeling at 14 d.p.i., supported by enhanced metabolic processes. Gene set 7 consists of 1,258 genes with an initial rise peaking at 7 d.p.i., involved in extracellular structure and collagen fibril organization during tendon repair.

Similarly, the differentially expressed genes in the aforementioned distinct sets were analyzed using STEM software, yielding similar results. Eight statistically significant gene clusters were subjected to GO analysis, where cluster 0 was linked to development and vascularization, cluster 1 to metabolism, cluster 2 to transcription and translation, cluster 5 to muscle development and differentiation, cluster 10 to extracellular matrix, cluster 23 to RNA processing, cluster 24 to stress, immunity, and inflammation, and cluster 26 to cell division and cell cycle. The trends and GO enrichment result observed by Mfuzz were in harmony with these results, thereby further confirming the consistency (Figure S4A).

Furthermore, to quantify the degree of infiltrating immune and stromal cells, we utilized xCell analysis. The enrichment scores of most immune cells at various time points post injury displayed similar changes, which is consistent with previous time-series analyses (Figure S4B).

### Cellular population composition in tendon repair following injury

**Figure 4:**
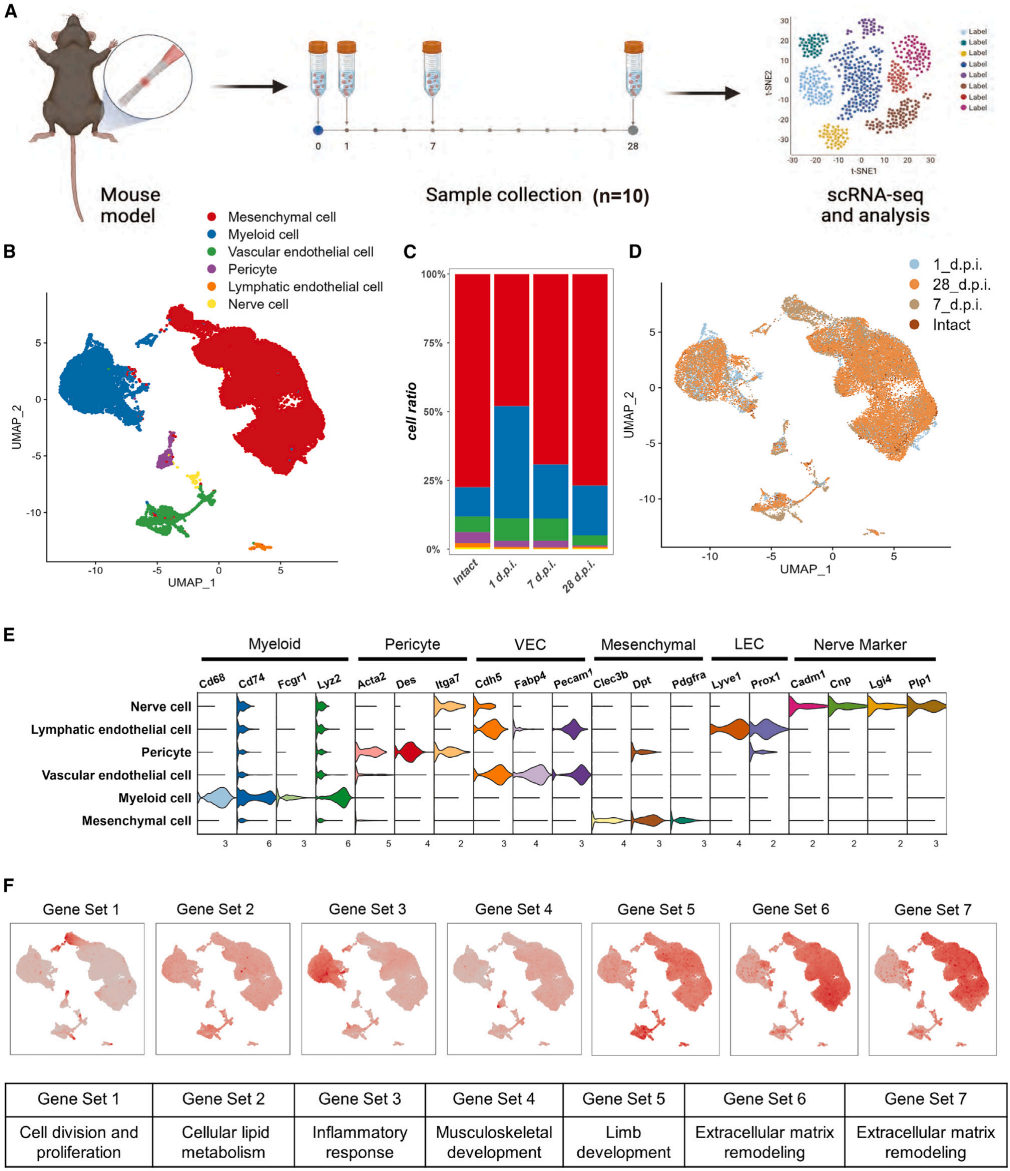
ScRNA-seq identified cellular heterogeneity in mouse tendon injury model (A) A graphical scheme of the Achilles tendon sampling process of scRNA-seq. (B) Dimension reduction UMAP plot based on lineage markers show the clustering result. (C) Relative proportions of different cell populations within each group. (D) The cell composition of each group. (E) Violin plot displaying the expression levels of different lineage marker genes. (F) The expression patterns of representative genes in the Mfuzz-clustered gene sets are visualized on the single-cell dimension reduction plot, with darker colors indicating higher expression level.

Bulk RNA-seq revealed seven distinct patterns of gene expression during the early stages of tendon repair. Notably, the most significant changes of gene expression for gene sets 2, 3, and 4 were observed at 1 d.p.i., while the most significant changes for gene sets 5 and 7 were observed at 7 d.p.i. Additionally, histological results indicated that cell infiltration was most prominent at 7 d.p.i. To investigate further, single-cell transcriptome sequencing was performed on normal tendon tissue, as well as on samples from the 1 d.p.i., 7 d.p.i., and 28 d.p.i. groups (Figure 4A). After excluding cells with low gene expression levels and those with high mitochondrial gene expression, a total of 30,507 cells from four time points were included, with 4,844 cells from the intact group, 5,060 cells from the 1 d.p.i. group, 9,176 cells from the 7 d.p.i. group, and 11,427 cells from the 28 d.p.i. group (Figures 4B and 4D).

According to their specific expression features, we have categorized the cell populations into six distinct groups (Figures 4B and 4E, Table S2). The mesenchymal cell group prominently expresses mesenchymal markers, such as Clec3b, Pdgfra, and Dpt.^4^ The myeloid cell population exhibits high expression levels of various myeloid markers, including Cd68, Fcgr1, Cd74, and Lyz2.^5^^,^^6^ The pericyte population shows high expression of Acta2, Itga7, Des, and other relevant biomarkers. The vascular endothelial cell population notably expresses Pecam1, Cdh5, Fabp4, and other known endothelial markers.^7^ The lymphatic endothelial cells display high expression levels of Lyve1, Prox1, and other specific lymphatic markers. Finally, the nerve cells demonstrate elevated expression of neuronal markers, including Plp1, Lgi4, Cadm1, and Cnp.^8^^,^^9^

In particular, our study reveals that there was a noticeable expansion of myeloid cells at 1 d.p.i. Subsequently, the proportion of myeloid cells decreases. Meanwhile, the proportion of mesenchymal cells steadily increased until 28 d.p.i., reaching a proportion similar to that in the intact group. However, the proportion of myeloid cells was still higher than that in the intact group (Figures 4C and 4D). This trend aligns with our previous analysis of immune cells using xCell. These findings underscore the complexity of the immune response to injury and suggest that myeloid cells and mesenchymal cells may play an important role in the repair process of tendon injuries.

Using the marker genes enriched in the GO terms of the seven gene sets as representative genes for scoring single-cell data, we observed distinct expression patterns across different cell groups (Figure 4F). The representative genes of gene set 1, associated with cell division and proliferation, are predominantly expressed in a subset of mesenchymal cells, with minimal expression in other cell groups. The representative genes of gene set 2, related to cellular lipid metabolism, show similar expression levels across all cell groups. The representative genes of gene set 3, linked to inflammatory responses, are highly expressed in myeloid cells. The representative genes of gene set 4, enriched in musculoskeletal development, are mainly expressed in mesenchymal cells and pericytes. Gene set 5 representative genes, enriched in limb morphogenesis, exhibit high expression in mesenchymal cells and also notably in vascular endothelial cells. The representative genes of gene sets 6 and 7, enriched in extracellular matrix organization, extracellular structure organization, collagen fibril organization, bone development, and glycoprotein metabolism, are primarily expressed in mesenchymal cells, with some expression observed in myeloid cells, vascular endothelial cells, and pericytes.

### Characteristics of the mesenchymal cell population

**Figure 5:**
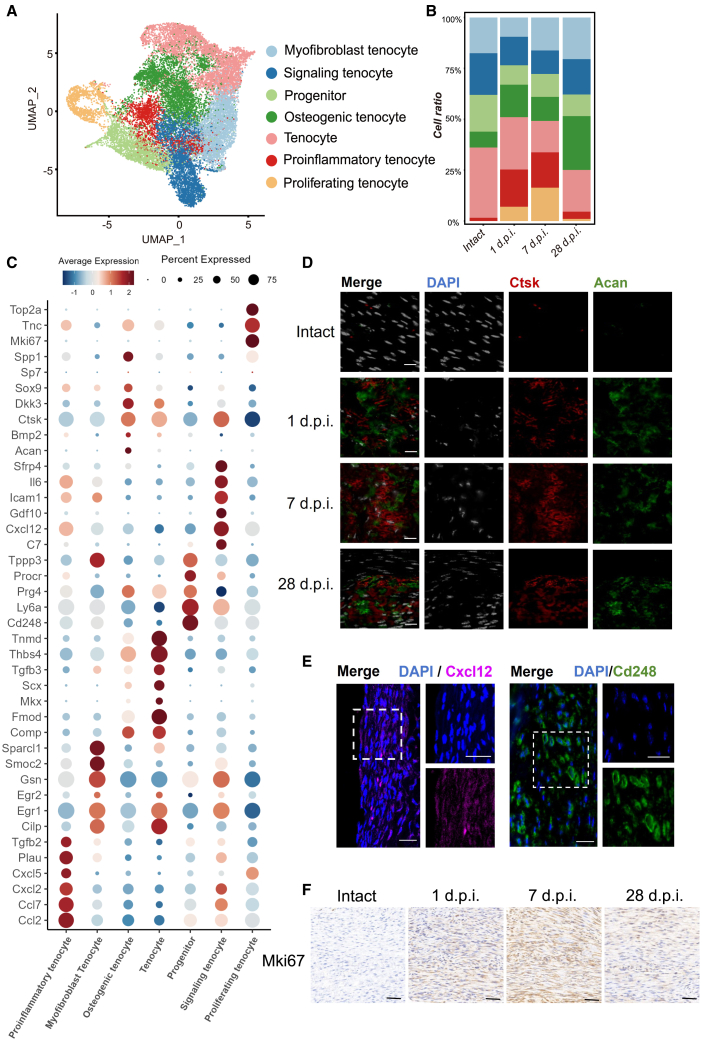
Characterization of mesenchymal cell subpopulations (A) The dimension reduction UMAP plot of mesenchymal cells, which can be further divided into seven distinct subgroups. (B) The distribution of individual cell subpopulations within the entire cell population of different time points. (C) Bubble plot illustrating the markers of mesenchymal cells, where redder colors represent increased expression levels, and larger circles indicate a higher percentage of cells expressing the gene. (D) Immunofluorescence of costaining of Ctsk and Aggrecan (Acan) revealed the existence of Ctsk+ osteogenic tenocyte in Achilles tendon. Scale bar:20μm. (E) Immunofluorescence verified the presence of certain mesenchymal cell subpopulation Cd248+ progenitor and Cxcl12+ signaling tenocyte in Achilles tendon. Scale bar:20μm. (F) Immunohistochemistry staining of proliferating tenocyte marker Mki67 at different time point post injury in Achilles tendon. Scale bar: 50μm.

To further investigate the subtypes and dynamic changes of mesenchymal cells during the early stages of tendon injury, we isolated mesenchymal cell and further categorized them into subgroups using dimension reduction techniques. By analyzing their differentially expressed genes, along with existing literature and biological knowledge, we manually annotated these distinct subsets of cells as progenitor, proinflammatory tenocyte, proliferating tenocyte, signaling tenocyte, osteogenic tenocyte, myofibroblast tenocyte, and tenocyte (Figure 5A). Next, we determined the proportions of different cell types at various time points. The most significant change was that tenocytes accounted for the largest proportion in the intact group, and their number decreases at 1 d.p.i. and 7 d.p.i. Later, their proportion will increase in 28 d.p.i. group. Osteogenic tenocytes were significantly increased at 28 d.p.i. and were present in the least proportion in the intact group. Myofibroblast tenocytes, the second most common cell type in 28 d.p.i. group, showed the sharpest decrease in 1 d.p.i. group, followed by an increase beginning on 7 days post-injury. Not surprisingly, proinflammatory tenocytes in 1 d.p.i. group and 7 d.p.i. group maintained high proportion, in accordance with the RNA-seq results described above. However, the highest proportion of progenitors was observed in the intact group. Whereas the proportion of progenitors decreased significantly in 1 d.p.i. group and remained at a low level in 7 d.p.i. group and 28 d.p.i. group. Proliferating tenocytes were also notably present in 1 d.p.i. group and 7 d.p.i. group and decreased in 28 d.p.i. group and intact group. Signaling tenocytes were distributed in similar proportions across all time points (Figure 5B).

According to the previously obtained differential expressed genes, we found that tenocytes highly express classic tendinous markers, including Scx, Fmod, Tnmd, and Thbs4.^10^ A cell subpopulation coexpressing tenogenic (e.g., Fmod and Thbs4) and osteochondrogenic markers (e.g., Ctsk and Acan), consistent with previous reports, were defined as osteogenic tenocytes.^11^^,^^12^^,^^13^ Proliferating tenocytes expressed a large number of genes related to cell proliferation, cell cycle and mitosis such as Mki67 and Top2a,^14^^,^^15^ suggesting that they are in an active state of cell proliferation. Progenitors had relatively high expression of Tppp3, Procr, Ly6a, and Cd248 in agreement with progenitors previously found in tendon, bone marrow and muscle.^16^^,^^17^^,^^18^ We also identified signature profiles of proinflammatory tenocytes (e.g., Cxcl5, Cxcl2, and Ccl7), myofibroblast tenocytes (e.g., Sparcl1, Cilp, and Col1a1), and signaling tenocytes (e.g., Cxcl12, Sfrp4, and Gdf10). All cell subpopulations were actively expressing their corresponding signature genes during different stages of tendon injury (Figure 5C). Furthermore, to validate the presence of the defined cell subpopulations, we performed immunofluorescence and immunohistochemistry staining on osteogenic tenocytes and proliferating tenocytes at various time points (Figures 5D and 5F). In addition, progenitors and signaling tenocytes at 28 d.p.i. were also subjected to immunofluorescence staining (Figure 5E).

**Figure 6:**
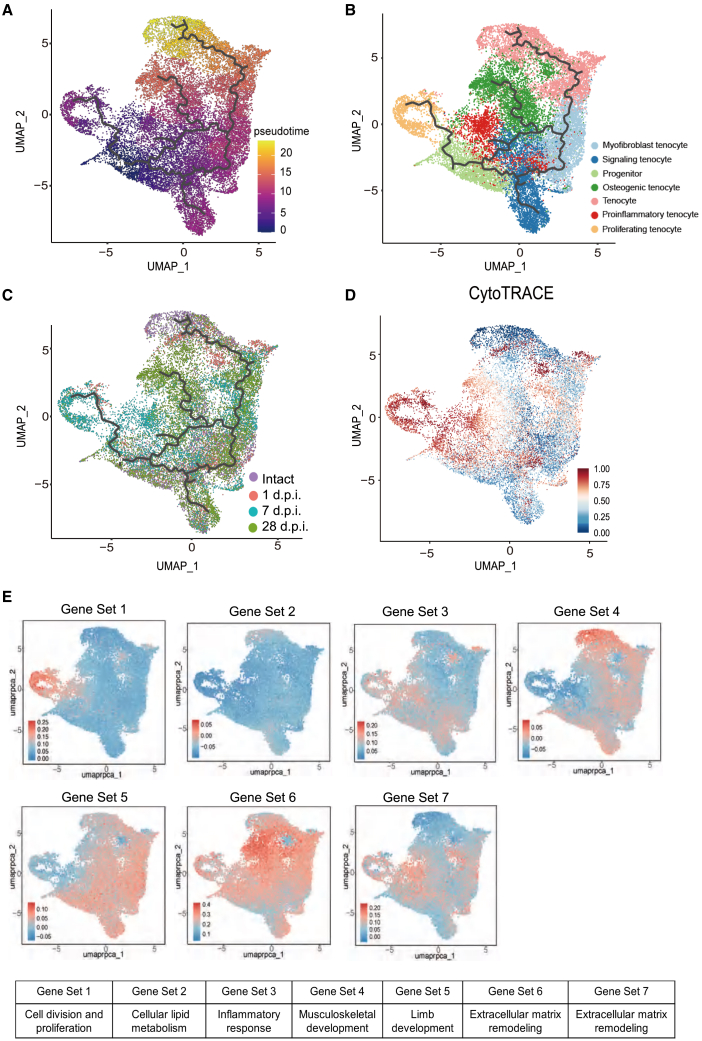
Trajectory and gene sets expression of the mesenchymal cell population (A) Pseudotime analysis reveal the process where a cell transit from an initial state to a final state. Lighter colors indicate that the cells are closer to the end of the transition trajectory. (B) Pseudotime trajectory based on cell clustering. (C) Pseudotime analysis based on different time points (intact, 1 d.p.i., 7 d.p.i., and 28 d.p.i.). (D) CytoTRACE evaluates the differentiation potential of cell populations derived from mesenchymal cells, with redder colors indicating higher levels of differentiation potential. (E) The expression patterns of representative genes in the Mfuzz-clustered gene sets are visualized on the mesenchymal cell UMAP, with redder colors indicating higher expression level.

To understand the differentiation and progression of the eight cell types, we conducted a pseudotime analysis on these cellular subsets using Monocle3.^11^ We illustrate the sequential arrangement of cells along the trajectory, starting from progenitors and transitioning to tenocytes (Figure 6A). The results revealed that the pseudotime trajectories of these eight cell types could be categorized into four major branches, all originating from progenitors. One branch directly differentiated into proliferating tenocytes, while another gave rise to signaling tenocytes. The third branch led to signaling tenocytes before ultimately culminating in osteogenic tenocytes. Notably, the journey to tenocytes passes through myofibroblast tenocytes (Figures 6B and 6C). Then, we used CytoTRACE to assess the differentiation potential of the eight mesenchymal cells. The analysis revealed higher prediction scores for progenitors, proliferating tenocytes, and proinflammatory tenocytes, which are represented by a darker red in the color gradient. Conversely, tenocytes, signaling tenocytes, as well as osteogenic tenocytes, exhibited relatively lower prediction scores and appeared lighter on the heatmap, suggesting a higher level of differentiation for these cell types (Figure 6D).

Subsequently, we evaluated the single-cell data of mesenchymal cells with previously obtained gene sets. Gene set 1, associated with cell division, represented genes primarily concentrated in proliferating cells and not expressed in the rest of the cell population. Gene set 2, representing genes related to the organic acid metabolism of lipid metabolism, showed relatively low expression across each cell population. Gene set 3, associated with inflammatory responses, was expressed in most cell subsets, particularly in proinflammatory tenocytes. Gene set 4 genes, enriched in muscle system processes, and gene set 5 genes, enriched in limb development and mesenchymal cell proliferation, showed similar distribution across each cell subpopulation, with gene set 4 genes being most strongly expressed in tenocytes. Gene set 6 GO terms, enriched in extracellular structure organization and extracellular matrix organization, showed high expression levels in all cell subsets, especially in osteogenic tenocytes and tenocytes. Gene set 7, associated with collagen fibril organization, bone development, and glycoprotein metabolism, showed enrichment in proinflammatory tenocytes (Figure 6E). Additionally, we analyzed the pseudotime expression of the genes of interest and observed that different genes exhibited distinct distribution patterns of dynamics along the pseudotime axis (Figure S5).

### Characteristics of the myeloid cell population

**Figure 7:**
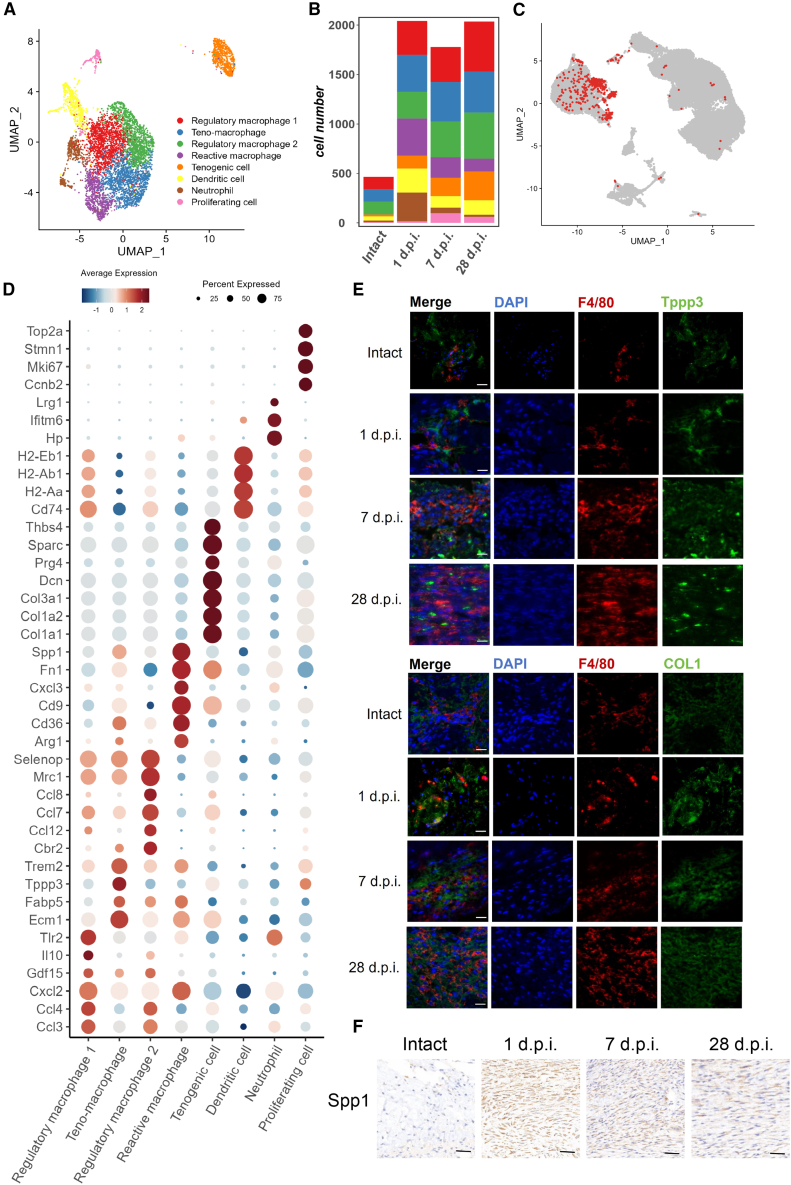
Characterization of myeloid lineage cell population (A) UMAP plot depicting the subsets within myeloid-derived cells, which can be further divided into eight distinct subpopulations. (B) Bar plot of cell number and proportion reveals the composition of each cell subpopulation across different groups. (C) Mapping tenogenic cells identified in myeloid lineage back to the original UMAP, with labeled cells represent as tenogenic cells, which mostly reside within myeloid cells. (D) Bubble plot illustrate the expression of marker genes of myeloid-derived cells. Darker color indicates higher expression, and larger circle represents a higher percentage of cells expressing the gene. (E) Immunofluorescence of costaining of myeloid marker F4/80 with teno-macrophage marker Tppp3 or tenogenic cell marker Col1 at different time point post Achilles tendon injury. Scale bar: 20μm. (F) Immunohistochemistry staining of reactive macrophage marker Spp1 at different time point post injury in Achilles tendon. Scale bar: 50μm.

Models of tendon injury typically exhibit inflammatory response post injury. RNA-seq analysis shows up-regulation of immune related genes from 1 d.p.i. to 28 d.p.i. Due to the essential role of myeloid cells in early tendon repair stages, our focus was on analyzing their specific cell subset. We performed dimension reduction analysis on myeloid cell populations, categorized them further into eight subsets based on differential gene expression. These subsets include reactive macrophage, regulatory macrophage 1 and 2, teno-macrophage, dendritic cell, tenogenic cell, neutrophil, and proliferating cell (Figure 7A). Neutrophils express neutrophil marker genes, such as Ifitm6, Lrg1, and Hp, and mostly present on day one after injury. Dendritic Cells (DCs) express dendritic cell signature genes, such as H2-Ab1, H2-Aa,^19^ and Cd74,^20^ which are distributed across various time points but are most frequently present at 1 d.p.i. (Figure 7B). Proliferating cells express genes associated with cell proliferation and mitosis, such as Top2a, Mki67, and Stmn1,^14^^,^^21^ peaking on day 7 post injury (Figure 7B). Tenogenic cells characterized by the expression of tenogenesis genes such as Col1a1, Col1a2,^22^ and Prg4,^4^ show a progressive increase in abundance, peaking at day 28 post-injury (Figure 7B). Apart from expressing classic monocyte/macrophage markers Cd14^5^ and Cd68,^23^ teno-macrophages also express tenogenic marker Tppp3^23^ and matrix protein Ecm1,^24^ suggesting they could be tendon-resident macrophage during tendon repair (Figures 7D and S6).

The combined immunofluorescence detection of myeloid marker F4/80^25^ and Tppp3 further confirms the possibility of the presence of teno-macrophages (Figure 7E). The combined immunofluorescence detection of myeloid marker F4/80 and Col1 further confirmed the presence of myeloid derived tenogenic cells (Figure 7E). And, the immunohistochemical staining of Spp1 at different time points confirmed the presence of reactive macrophages (Figure 7F). Reactive macrophages take large part of the total count within one week after injury and gradually decrease with the process of healing. Given the expression of specific inflammatory genes and the temporal changes involved in the repair process, this group of cells may be involved in the activation of early inflammatory responses and the regulation of the inflammatory microenvironment during recovery.^2^ The two groups of regulatory macrophage subsets exhibited distinct characteristics during the tendon repair process, with the highest numbers observed on the 28th day post injury. Regulatory macrophage subset 1 overexpresses immune-regulating genes such as Tlr2^26^ and Il-10,^27^ suggesting a role in regulating the immune microenvironment following tendon injury. In contrast, regulatory macrophage subset 2 is highly expressed in macrophage regulatory and chemokine-related genes, such as Mrc1,^28^ Ccl7 and Ccl8,^29^ indicating its involvement in the regulation of inflammatory response cells, primarily macrophages, after injury.

**Figure 8:**
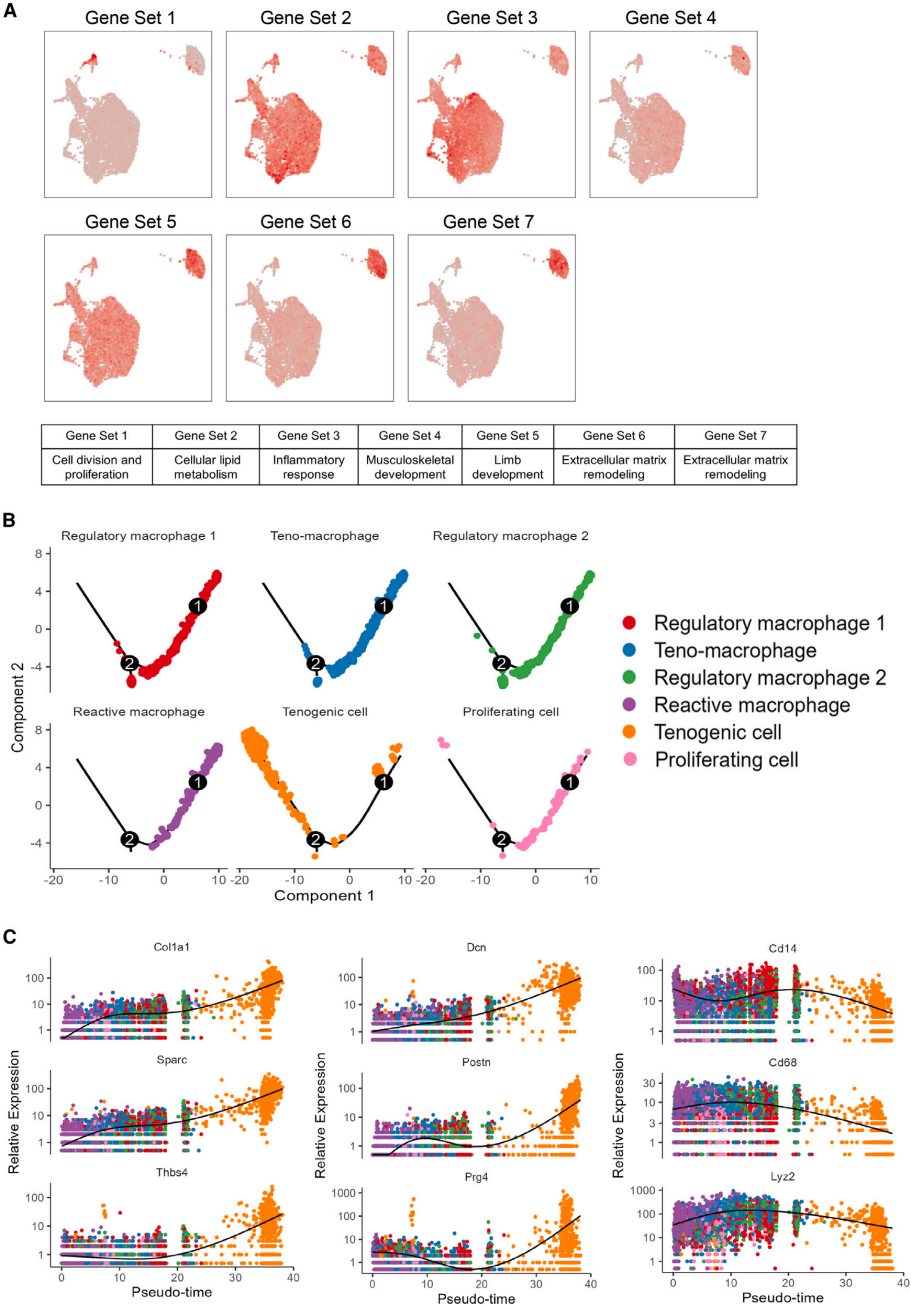
Trajectory and gene sets expression of the myeloid cell population (A) The expression patterns of representative genes in seven Mfuzz-clustered gene sets are visualized on the myeloid cell UMAP, with redder colors indicating higher expression level. (B) Distribution pattern of each cell subpopulation along a pseudotime cell trajectory. (C) The relative expression of tenogenesis related genes, such as Dcn, Postn, Prg4, Col1a1, Sparc, and Thbs4, exhibit a gradual increase as the pseudotime axis approaches the tenogenic cell. Meanwhile, the relative expression of macrophage related genes, such as Cd14, Cd68 and Lyz2 exhibit a gradual decrease as the pseudotime axis approaches the tenogenic cell.

Then, we employed a similar strategy to match the single-cell data of myeloid cells using the representative gene set. We identified that the representative genes in gene set 1, associated with cell division, were enriched in proliferating cells. The genes in gene set 2, linked to organic acid metabolism and lipid metabolism, showed moderate expression across every cell population. Inflammation-associated genes constituted gene set 3 were expressed in most subpopulations of myeloid cells, particularly in regulatory macrophages 1, reactive macrophages, and neutrophils. Gene set 4 genes, characterized by enrichment in muscle system development, showed low expression in all subgroups. Limb development and mesenchymal cell proliferation related gene set 5 were especially evident in tenogenic cell subsets. During the late stages of tendon repair, myeloid-derived tenocytes may promote tissue repair, reducing inflammation and promoting tissue reconstruction by producing anti-inflammatory cytokines such as IL-10 and TGF-β.^30^ The representative genes in gene set 6 and 7 enriched in GO terms such as extracellular structure organization, extracellular matrix organization, bone development, and glycoprotein metabolism exhibited high expression levels in tenogenic cell populations (Figure 8A).

Considering the potential of myeloid cells transforming into fibroblast-like cells and their close association with fibrosis, we conducted Monocle2 pseudotime analysis^31^ on myeloid-derived subsets. Since tenogenic cells deviated from the primary cell population in the UMAP reduction map of myeloid cells, we re-mapped this population of tenogenic cells back into the original UMAP. Majority of tenogenic cells were dispersed within myeloid cell populations (Figure 7C). This could be due to the greater weight of tendon-related genes in mesenchymal cells in the original UMAP, with the primary markers of tenogenic cells derived from macrophages being myeloid origin markers. In the second-dimension reduction of myeloid lineage cells, tendon-related genes exhibited a discernable impact on their positions. When these myeloid populations were distributed on the cell trajectory by Monocle2, most macrophage subsets and proliferating cells were on one side of the trajectory, while tenogenic cells were on the other side (Figure 8B). Notably, tendon-related genes, including Col1a1, Sparc, Thbs4, Dcn, Postn, and Prg4, displayed on the pseudotime axis, demonstrated a gradually increased relative expression levels as the pseudo timeline approaching tenogenic cells.^32^^,^^33^ Meanwhile, the relative expression levels of myeloid-associated genes, including Cd14, Cd68, and Lyz2, gradually decreased as the timeline approaching tenogenic cells, indicating the potential transition process of myeloid cells to tenogenic cells (Figure 8C).

## Discussion

Dissecting the molecular programs that underlie the physiological and pathological aspects of early tendon healing is critical for developing and translating biological and pharmacological approaches to improve tendon healing. Given the lack of early diagnosis standards and sufficient sample numbers in clinical settings, we sought to establish a reliable puncture-induced tendon injury mouse model to study the overall early healing process. By utilizing transcriptome sequencing techniques, we successfully mapped out the molecular and cellular dynamics associated with early tendon injury.

Compared with normal tendons, we observed varying degrees of enrichment in genes related to leukocyte migration, chemotaxis, leukocyte activation, and positive response to external stimuli at different time points during early tendon injury. Similar to the conclusions of previous research, on the 1 d.p.i. , 3 d.p.i., and 7 d.p.i., tendons may primarily activate and sustain the inflammatory microenvironment in the damaged area through IFN-mediated interferon signaling pathways and IL-1-mediated NF-κB signaling pathways.^34^^,^^35^ Consistent with gene expression, the GO terms indicated a significant presence of inflammation at 1 d.p.i. This phenomenon can be attributed to the significant influx of leukocytes from the blood or surrounding tissues toward the injured site.^36^ On the third day, genes associated with the inflammatory response remained present, while simultaneous changes in lipid metabolism processes were observed. On the seventh day post injury, we observed persistent inflammation alongside regulatory processes related to skeletal system differentiation. This suggests potential tissue repair mechanisms following tendon injury. The observed phenomena may be attributed to the activation and differentiation of stem cells induced by inflammatory factors and growth factors.^37^^,^^38^ Somewhat surprisingly, the genes that were upregulated at 28 days were mainly enriched in GO terms related to the composition of the extracellular matrix, mechanisms of cell adhesion, cytokine production, and metabolic processes. This observation suggests that tendon cells undergo increased synthesis of the extracellular matrix to facilitate the remodeling of tendon structure and restoring function following injury.

Then, we found that mesenchymal cell differentiation in the tendon occurred in four main directions, with one pathway ultimately leading to the formation of osteogenic tenocytes. This finding is consistent with the common clinical phenomenon of heterotopic ossification following tendon injury.^39^ Another branch passes through the myofibroblast tenocytes and eventually differentiates into tenocytes (Figures 6A and 6B). Simultaneously, we observed that the majority of tenocytes remained grouped in the intact group, whereas myofibroblast tenocytes are predominantly found in the 28 d.p.i. group, indicating the occurrence of fibrosis in the tendon which prevents it from returning to its original normal state. These observations appear to correlate with the transition from thick fibers (intact) to thin fibers (28 d.p.i.) observed in transmission electron microscopy (Figure 1F). Throughout different stages of tendon repair, the expression levels of various members of the TGF-β family change. In the early stage (7 d.p.i.), there is a slight increase in TGF-β2 expression, whereas at the later stage (28 d.p.i.), TGF-β3 expression shows a significant rise. These changes underscore the regulatory role of TGF-β in tendon development and repair, particularly in the formation of collagen structures. TGF-β2 is expressed in tenocytes and regulates collagen production through the TGF-β signaling pathway, which may contribute to irregular collagen structure formation.^40^ In contrast, TGF-β3 expression in tenocytes inhibit TGF-β binding to its receptor, promoting the formation of organized collagen structures. These findings emphasize the critical role of the TGF-β family in tendon repair and functional recovery.

Our work further highlighted specific teno-macrophages expressing Tppp3 and macrophage-associated markers. Additionally, these cells also express Fabp5 and Trem2, which are associated with macrophage polarization and fibrosis.^41^ We speculate that teno-macrophages may be specialized tendon-resident macrophages that regulate tendon homeostasis and repair processes through secrete factors such as chemokines, signaling pathway regulators, and immune complement. Additional experiments (e.g., ChIP-seq, gain- and loss-of-function transgenic mouse models) are needed to determine their roles for regulating early tendon injury. Moreover, we also identified specific tenogenic cells from myeloid cell lineage that highly express tendinous genes, such as Col1a1, Col1a2, Thbs4, and Prg4, at different time points during tendon repair. We speculate that this subtype of myeloid-derived cell may have undergone a macrophage-to-tenocyte transition during the repair process of tendon injury, thereby participating in tendon repair. But further work needs to be performed to better evaluate the contribution of tenogenic cells and whether the cells function during healing via a cell-autonomous or non-cell-autonomous mechanism.

In summary, our data provide unprecedented cellular and molecular profiling of the early tendon healing process and defining the molecular programs underlying distinct time points. Recognizing the specific molecular programs and cellular subpopulations predominant during different healing phases will substantially enhance our understanding of the complex cellular and molecular milieu of tendon healing, facilitating the identification of more targeted therapeutic approaches to improve tendon repair.

### Limitations of the study

Although this study effectively models the early progression of tendon injury, it is important to acknowledge several potential limitations. First, no further relevant experimental verification was conducted. Then, due to the limited availability of samples, our study exclusively utilized mice as subjects. Therefore, future investigations should incorporate human samples in order to elucidate more precise molecular mechanisms underlying tendon injury. Finally, the constructed mouse tendon injury model may represent early tendon disease, but further validation is needed.

### Conclusions

In conclusion, we propose the dynamic changes of molecular pattern and cellular subpopulation in a puncture-induced tendon injury model. Our study identifies seven gene sets with specific expression profiles, eight types of myeloid-lineage cells, and seven types of mesenchymal-lineage cells during the early tendon injury process. Development of therapeutic strategies focused on teno-macrophages, and tenogenic cells may have a potential application to various tendon injuries.

## Resource availability

### Lead contact

Further information and requests for resources and reagents should be directed to and will be fulfilled by the lead contact, Weiliang Shen (wlshen@zju.edu.cn).

### Materials availability

This study did not generate new unique reagents.

### Data and code availability

• Data: The RNA-Seq, and scRNA-seq data are deposited in the Gene Expression Omnibus (GEO): GSE288443, GSE288444. Further information and requests for any data reported in the manuscript should be directed to the corresponding author Weiliang Shen (wlshen@zju.edu.cn).
• Code: This study did not generate any original code.
• Any additional information required to reanalyze the data reported in this paper is available from the lead contact upon request.

## Acknowledgments

This research was supported by 10.13039/501100004731Zhejiang Provincial Natural Science Foundation of China (ZCLQN25H0601), 10.13039/501100001809NSFC grants (82372376, T2121004, 32271406), Zhejiang “Lingyan” Research and Development Project (2024C03207, 2024C03077), Zhejiang Provincial Program for the Cultivation of High-level Innovative Health talents, Dr Li Dak Sum & Yip Yio Chin Regeneration Medicine Foundation.

## Author contributions

W.S., X.C., and J.J. designed the study. Z.H. and D.R. conducted the experiments. H.C., Y.F., C.W., and J.H. acquired samples and assisted with the experiments. Z.H., Z.L., and Y.X. analyzed the data. The manuscript was written by Z.L., D.R., and H.J. and reviewed by all authors. All authors read and approved the final manuscript.

## Declaration of interests

The authors declare no competing interests related to this work. All research was conducted independently, without any influence from funding bodies, organizations, or commercial entities.

## STAR★Methods

### Key resources table

**Table:** 

REAGENT or RESOURCE	SOURCE	IDENTIFIER
**Antibodies**		

CTSK Rabbit pAb	ABclonal	RRID: AB_2766620; Cat#A5871
Aggrecan Monoclonal Antibody (BC-3)	Thermo Fisher Scientific	RRID: AB_568440
CXCL12 Rabbit pAb	ABclonal	RRID: AB_2862001; Cat# A18225
CD248 Polyclonal Antibody	Thermo Fisher Scientific	RRID: AB_2899612
Purified anti-mouse/human Ki-67 Antibody	Biolegend	RRID: AB_2566621
Purified anti-mouse F4/80 Antibody	Biolegend	RRID: AB_893504
TPPP3 Polyclonal Antibody	Proteintech	RRID: AB_2878105; Cat# 15057-1-AP
Osteopontin Polyclonal Antibody	Thermo Fisher Scientific	RRID: AB_795087
Collagen I Antibody	Affinity	RRID: AB_2835309; Cat# AF7001
Alexa Fluor 647-labeled Goat Anti-Rabbit IgG(H + L)	Beyotime	RRID: AB_2936379; Cat# A0468
Alexa Fluor 647-labeled Goat Anti-mouse IgG(H + L)	Beyotime	RRID: AB_2891322; Cat# A0473
Anti-Rabbit IgG H&L (Alexa Fluor® 488)	Abcam	RRID: AB_2630356; Cat# ab150077
Anti-Mouse IgG H&L (Alexa Fluor® 555)	Abcam	RRID: AB_2687594; Cat# ab150114
ABflo® 488-conjugated Goat anti-Mouse IgG (H + L)	ABclonal	RRID: AB_2768319; Cat# AS037

**Biological samples**		

Mouse tendon	This paper	N/A

**Critical commercial assays**		

NEBNext® UltraTM RNA Library Prep Kit	Illumina	Cat#E7530
GEXSCOPE® Single Cell RNA Library Kit	Singleron	SD: 4180011

**Deposited data**		

Raw and analyzed data	This paper	The RNA-Seq, and scRNA-seq data are deposited in the Gene Expression Omnibus (GEO):GSE288443, GSE288444.
Mus musculus reference genome, GRCm39	Ensembl.org	https://ftp.ensembl.org/pub/release-105/gtf/mus_musculus/

**Experimental models: Organisms/strains**		

Mus musculus C57BL/6N strain	Charles river	https://www.vitalriver.com/rm-find-model/213-c57bl-6n.html

**Software and algorithms**		

R	The R Foundation for Statistical Computing	https://www.r-project.org/
FastQC	N/A	https://www.bioinformatics.babraham.ac.uk/projects/fastqc
Fastp	Chen et al.^15^	https://github.com/OpenGene/fastp
STAR	Dobin et al.^42^	https://github.com/alexdobin/STAR
featureCounts	Liao et al.^43^	https://subread.sourceforge.net/
DESeq2	Love et al.^44^	http://www.bioconductor.org/packages/release/bioc/html/DESeq2.html
clusterProfiler	Wu et al.^45^	https://bioconductor.org/packages/release/bioc/html/clusterProfiler.html
Mfuzz	Kumar et al.^46^	http://itb1.biologie.hu-berlin.de/∼futschik/software/R/Mfuzz/index.html
STEM	Ernst et al.^47^	https://www.cs.cmu.edu/∼jernst/stem/
Monocle 2	Qiu et al.^31^	https://github.com/cole-trapnell-lab/monocle-release
Monocle 3	Cao et al.^11^	https://cole-trapnell-lab.github.io/monocle3/

### Experimental model and study participant details

All experiments were performed with the approval of the Institutional Animal Care and Use Committee of the Zhejiang Center of Laboratory Animals (Grant No: ZJCLA-IACUC-20050062, Date of approval: 2021.11.29 and No: ZJCLA-IACUC-20010700; Date of approval: 2024.3.24). All animal experimentation conformed to the Guide for the Care and Use of Laboratory Animals. 8–10 weeks old male and female C57BL/6N mice were used for all experiments. All mice were kept on *ad-libitum* (AL) access to food and tap water, and kept under standard housing conditions, with 12h light/12h dark cycles, 30–70% humidity and a temperature of 20°C–23°C unless specified otherwise.

### Method details

#### Surgical models

The multipoint needling device consists of a fixed platform and multiple needles, nine needles is evenly closed in a circle, distributed in multiple rows, and manufactured by LiAo tech (Hunan province, China). In this study, we maintained a constant depth and frequency of acupuncture to ensure the standardization and reproducibility of the treatment procedure for each mouse. Mice were anesthetized prior to surgery. After cleaning and disinfecting the surgical area, the tendon was exposed, and an ophthalmic forceps was placed beneath the tendon to support the multi-row needling procedure. The tendon was then punctured using the multipoint needling device. After surgery, the skin was closed with a 5-0 suture.

#### Histological evaluation

Upon the euthanasia of mice, tendon was harvested and fixed in formalin 4% (vol/vol) for 48 h, processed and embedded in paraffin. Sagittal 7μm-thick sections were cut parallel to the long axis of tendon, placed on glass slides and stained with hematoxylin and eosin. All sections were read by 2 blinded individual investigators based on a modification of the Movin grading system (Table S1).^15^ Six parameters were evaluated on a 0–3 scale, with 0 being least abnormal and 3 being most abnormal.

#### Bulk RNA sequencing (RNA-seq) and differentially expressed genes (DEGs) analysis

To ensure sufficient mRNA extraction amount, 4 patellar tendons from every 4 mice in the same group were combined together as one sample, and histological samples were obtained at 6 time points. Day 0 (intact group), day 1, day 3, and day 7 groups each had 4 samples; day 14 and day 28 groups each had 5 samples. After harvesting, the tissues were immediately placed into liquid nitrogen and then ground. Total RNA was isolated by TRIzol Reagent (Thermo Fisher Scientific, USA), mRNA was purified using poly-T oligo-attached magnetic beads, fragmented and then reverse-transcribed into double-stranded cDNA. A sequencing library was generated using NEBNext UltraTM RNA Library Prep Kit for Illumina (NEB, USA) and the clustering of index-coded samples was generated using TruSeq PE Cluster Kit v3-cBot-HS (Illumia, USA). Sequencing was performed on a HiSeq Novaseq platform (Illumina). The RNA-seq raw reads were processed with fastQC (0.11.9) and fastp (0.12.4) to remove low quality reads.^36^ Readings were aligned to the reference genome Mus musculus.GRCm39.105 using STAR (2.7.10a)^42^ and gene counts were calculated by featureCounts^43^ from the Subread package (2.0.1). The R (4.2.2) was used for subsequent analysis for all sequencing data. DESeq2 (1.34.0)^44^ was utilized to determine the DEGs between groups with Benjamini and Hochberg methods adjusting *p* value to control false discovery rate (FDR). R package clusterProfiler (4.2.2)^45^ were used to perform Gene Ontology (GO) analysis and GO terms with adjusted *p* < 0.05 were deemed as significant.

#### Temporal analysis of transcriptomic data by Mfuzz and STEM

The DEGs obtained from different pairs were integrated. After integration, the R package Mfuzz (2.54.0)^46^ and STEM software (1.3.13)^47^ were used for analysis. Mfuzz packages used fuzzy c-means clustering to identify gene profiles with similar expression characteristics. The integrated data is imported and standardized by the functions included in the package. R package fviz_nbclust and factoextra were used to determine and visualize the optimal number of clusters. After determining the optimal number, Mfuzz were utilized to get the clustered gene sets. STEM is a Java program that clusters, compares, and visualizes gene expression data at short time points. After the DEGs with TPM normalization were imported, the analysis proceeds according to the software instruction. Similarly, clusterProfiler (4.2.2) were conducted to obtain GO enrichment results.

#### Single cell RNA sequencing

##### Tissue dissociation

Based on the results of the bulk RNA-seq, we selected four time points for single-cell transcriptome sequencing, including the intact group, 1 day post-injury group (1 d.p.i.), 7 days post-injury group (7 d.p.i.), and 28 days post-injury group (28 d.p.i.). To ensure a sufficient number of single cells, we pooled 10 Achilles tendons from 10 mice at the same time point as one sample for processing. Upon the euthanasia of mice, the mice were perfused with normal saline and tendons were dissected and placed in Dulbecco’s modified Eagle’s medium (low glucose; Thermo Fisher Scientific, USA) with 10% (vol/vol) fetal bovine serum (FBS, Gibco, USA) and 1% (vol/vol) penicillin-streptomycin (Thermo Fisher Scientific, USA). The specimens were rinsed twice with phosphate buffered saline (PBS) and minced, then subjected to pre-warmed dissociation solution (3 mg/mL Collagenase I (Gibco, USA) and 4 mg/mL Dispase II (Roche, Swiss) at 37°C, 5%CO_2_ cell incubator for 2 h. Every 30 min the tissues were gently blown by pipette. After digestion, 40-micron sterile strainers (Biosharp, China) were used to filter residue and the samples were centrifuged at 1,200 rpm for 5 min. To obtain single cell suspension, supernatant was discarded and 0.5mL PBS were added to resuspend the sediment. The cell viability and number were evaluated by Countstar cell counter (Countstar, China).

##### Sequencing and data processing

Single-cell suspensions (1×10^5^ cells/mL) with PBS were loaded onto microwell chip using the Singleron Matrix Single Cell Processing System. Barcoding beads collection, mRNA transcription, PCR amplification, fragmentation, ligation and library construction were conduct as per manufacture’s protocol (GEXSCOPE Single Cell RNA Library Kits, Singleron, China). Individual libraries were diluted to 4 nM, pooled, and sequenced on Illumina novaseq 6000 with 150 bp paired end reads. Quality control, read mapping, gene counts and unique molecular identifier (UMI) counts were processed as described in 2.3 by the celescope pipeline of singleronbio. Matrix files were generated based on gene counts and UMI count.

#### Transmission electron microscopy

Fresh tendon samples were meticulously dissected and immediately immersed in 2.5% glutaraldehyde (dissolved in 0.1M phosphate buffer) overnight and then underwent a series of through rinsing with phosphate buffer for 15 min per wash. Subsequently, the samples were subjected to a secondary fixation using 1% OsO4 (dissolved in 0.1M phosphate buffer), for 1 h. This was followed by a similar washing procedure to remove excess fixative.

After fixation, samples were systematically dehydrated in a gradient concentration of ethanol (30%,50%, 70% and 80%) for 15 min at each step, which was succeeded by further dehydration using a gradient concentration of acetone (90% and 95%) for 15 min each. Then, the samples were dehydrated twice by absolute acetone for 20 min respectively.

The specimen was infiltrated with mixture of absolute acetone and the final Spurr resin in a 1:1 ratio for 1h at room temperature, and then placed in mixture of absolute acetone and the final resin in a 1:3 ratio for 3 h. Subsequently, the specimen was transferred to pure Spurr resin overnight.

Finally, specimen was placed into eppendorf tubes containing Spurr resin and was heated at 70°C for 12 h. The hardened specimens were sectioned using LEICA EM UC7 ultramicrotome. The resulting sections were stained with uranyl acetate and alkaline lead citrate for 5 and 10 min, respectively. The ultrastructure of tendon was examined using a Hitachi Model H-7650 transmission electron microscope.

#### Microcomputed tomography

Microcomputed tomography (mCT, Bruker SkyScan 1276) with an energy of 55 kV peaks, an intensity of 145 mA, and a standard resolution of 5 mm was used to scan samples. After image reconstruction, region of interest around the injury region was selected by a rectangle with a fixed dimension. For 3D visualization, the reconstructed images were imported into CTVOX (Bruker).

#### Immunofluorescence

Mouse patellar tendon tissue slices were deparaffinized and hydrated. Then antigen retrieval was performed with sodium citrate antigen retrieval solution (Solarbio, China) at 60°C overnight. The next day slides were blocked with QuickBlock blocking buffer (Beyotime, China) for 30 min and treated with primary antibodies at 4°C overnight. After washed by PBST for 3 times, the slides were incubated with second antibodies at room temperature for 1 h and cell nuclei were stained with DAPI solution (Solarbio, China), which were later imaged with a fluorescence microscope (Leica S5, Germany). For co-staining, after incubation with second antibodies, slides were washed with PBST for 4 times, blocked again with blocking buffer and treated with another primary antibodies at 4°C overnight.

### Quantification and statistical analysis

All quantitative data were analyzed with the use of R software, version 4.2.2. Histological results were expressed as mean ± standard deviation (Mean ± SD). The Shapiro test was also used to test the normality of the histological scores of multiple groups of samples. Because the histological scores did not meet the normal distribution assumption, Mann-Whitney U tset was used for analysis. *p* < 0.05 was used as the threshold for statistical significance. Statistical plots were made using the R package "ggplot2".
