# Supplementary material for: Dynamic changes of molecular pattern and cellular subpopulation in puncture-induced tendon injury model
Source: iScience. 2025 Feb 19;28(4):112034. doi: 10.1016/j.isci.2025.112034 (PMC11994932; doi:10.1016/j.isci.2025.112034)
Supplement: Document S1. Figures S1–S6 and Tables S1 and S2 [file mmc1.pdf]

iScience, Volume *28*

Supplemental information

Dynamic changes of molecular pattern and cellular subpopulation in puncture-induced tendon injury model

Zizhan Huang, Ziyang Li, Dengfeng Ruan, Yiwen Xu, Honglu Cai, Hengzhi Liu, Haocheng Jin, Peiwen He, Yang Fei, Jiayun Huang, Canlong Wang, Xiao Chen, Jia Jiang, and Weiliang Shen

Supplementary

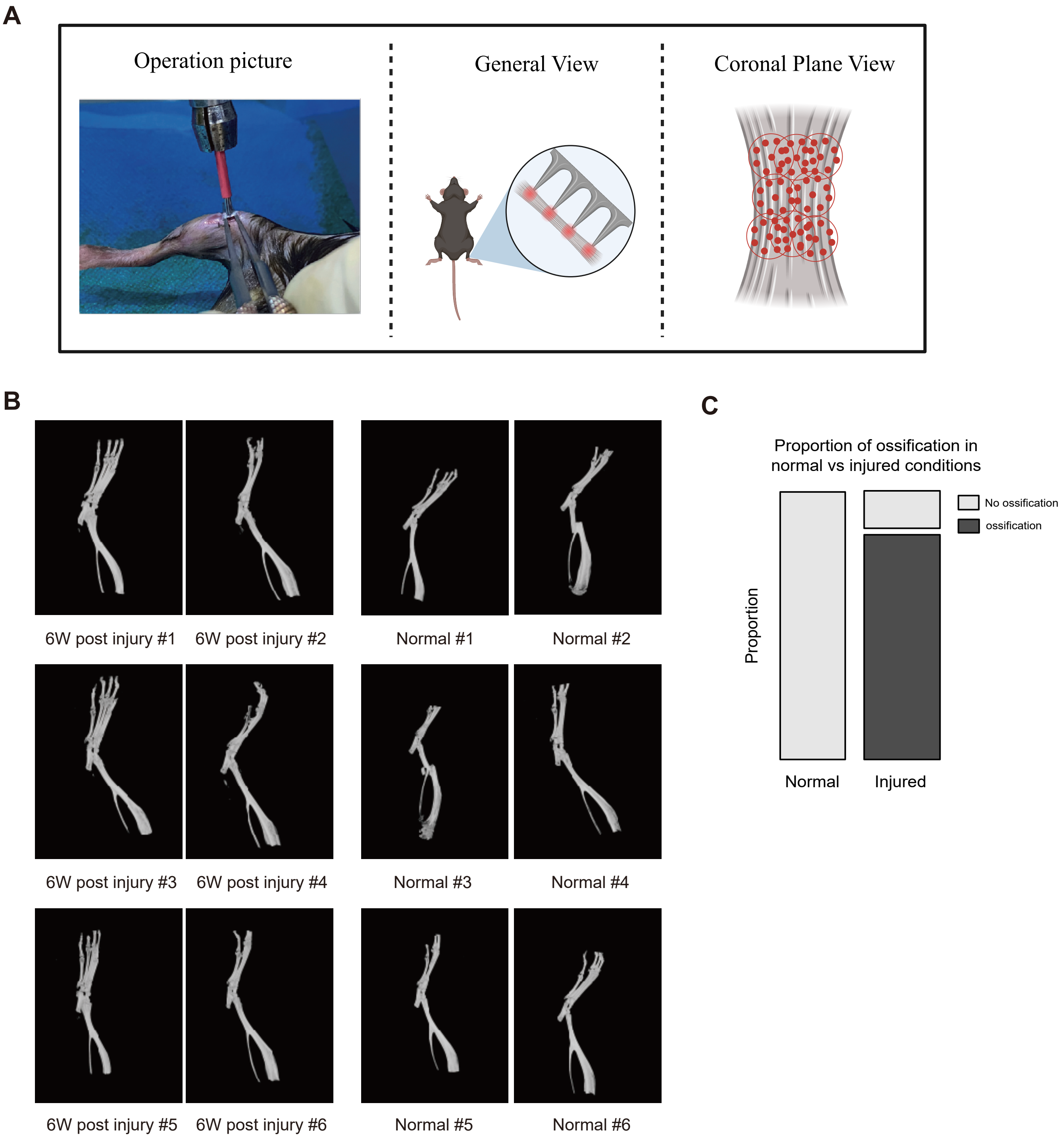

Figure S1. Views of puncture-induced tendon injury model and the tendon calcification post injury, related to Results and Figure 1.

(A) A schematic diagram demonstrating the details of puncture process.

(B) Micro CT reveals the existence of heterotopic ossification after 6 weeks of puncture-induced injury in Achilles tendon.

(C) The proportion of calcification level of normal and injured Achilles tendons.

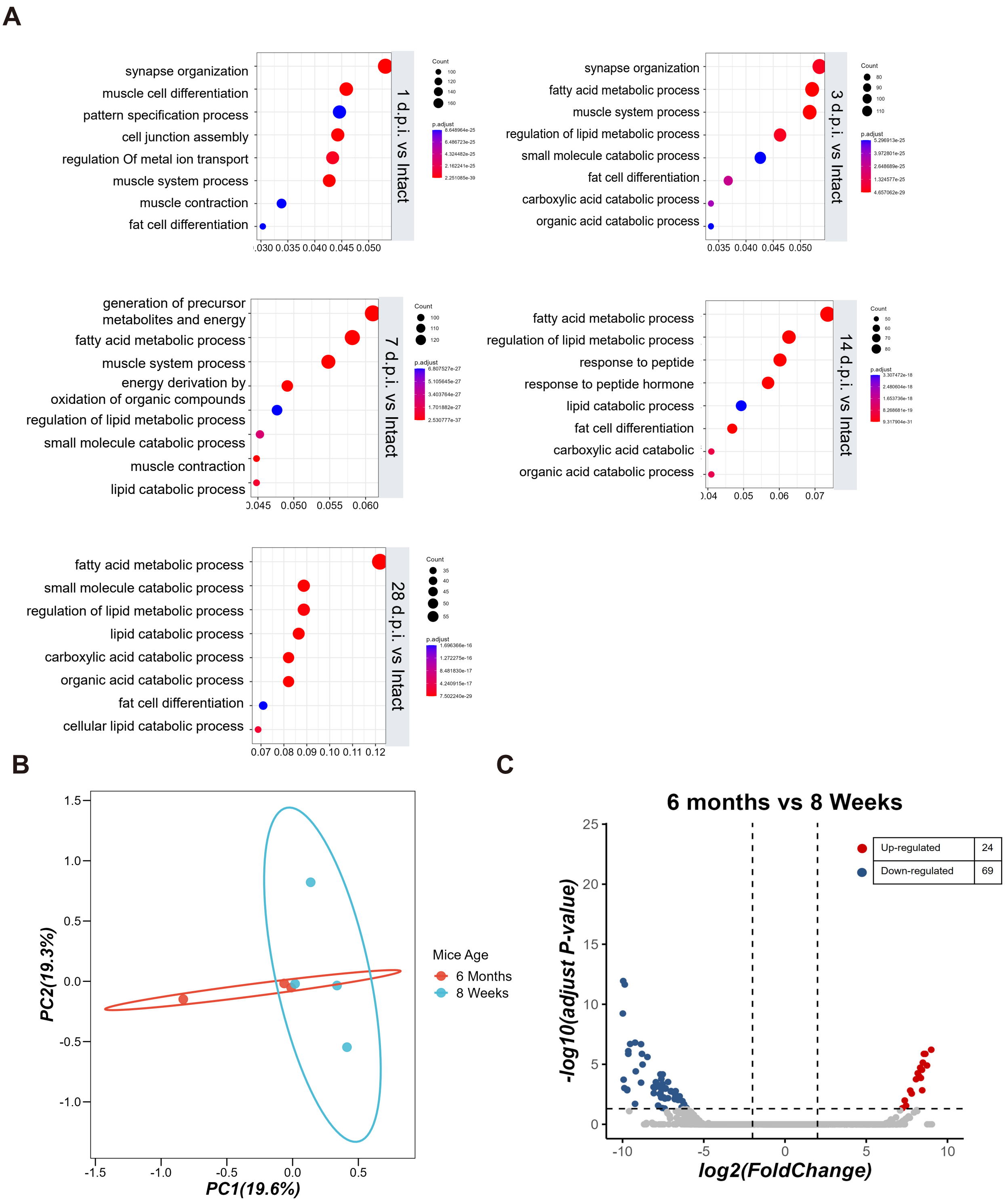

Figure S2. GO enrichment of DEGs between injured and uninjured groups and the comparison between 6 months and 8 weeks mice tendon, related to Results and Figure 2.

(A) Enrichment of down-regulated genes in Gene Ontology (GO) biological process terms obtained by pairwise comparisons between the injured and uninjured groups (log_2foldchange ≤-1, adjusted p value <0.05). Top GO terms were determined based on the adjusted p value.

(B) Principal component analysis of RNA-seq result of 6 months old mice patellar tendon versus 8 weeks old mice patellar tendon shows overlap and neither PC1 nor PC2 could clearly separate the data.

(C) Volcano plot for differentially expressed genes between 6 months old mice patellar tendon and 8 weeks old mice patellar tendon (|log2FoldChange| ≥1, adjusted p value <0.05). 24 genes were upregulated and 69 genes were downregulated, indicating their high transcriptional similarity.

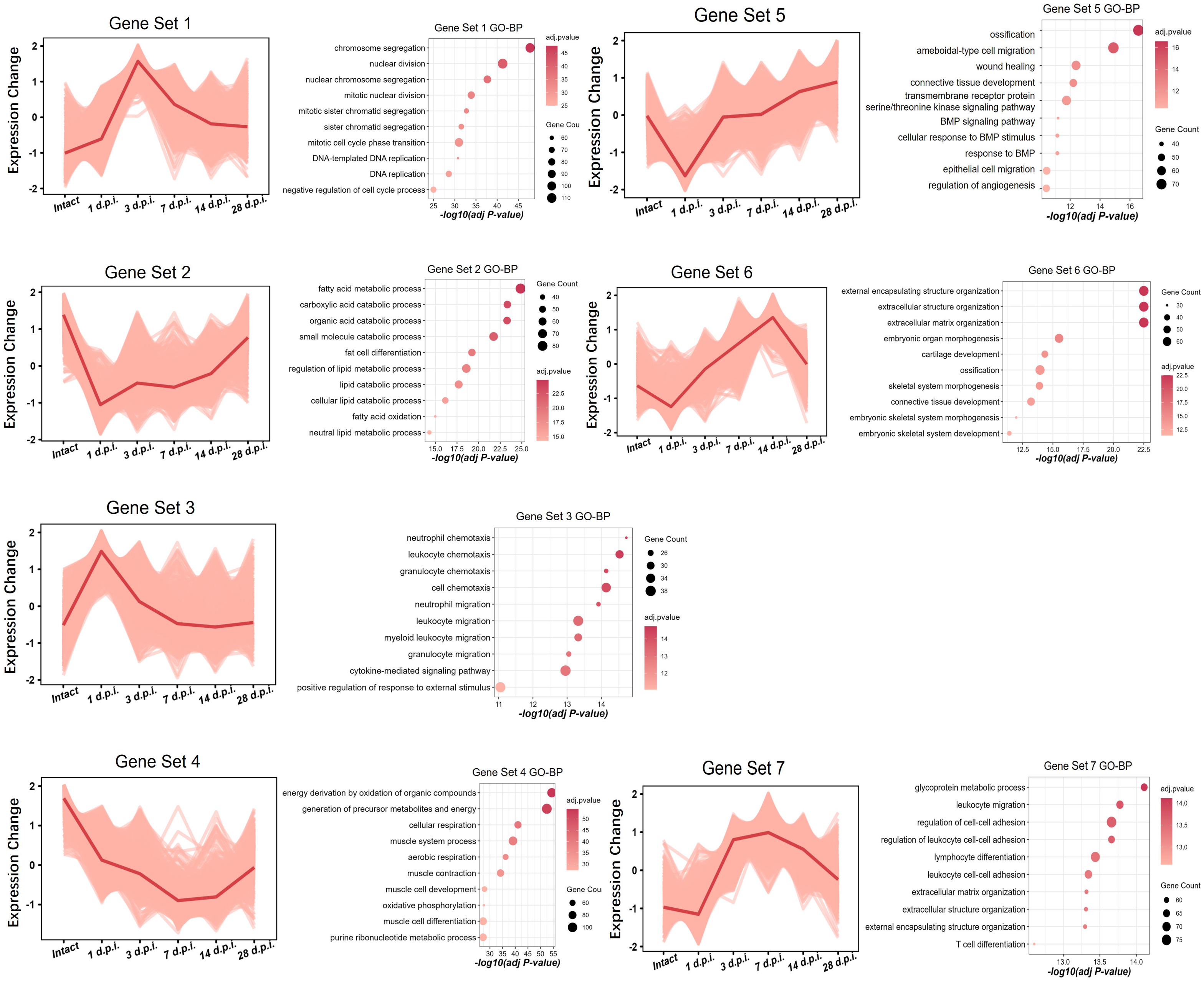

Figure S3. The Mfuzz-clustered DEGs expression trends of female mice post tendon injury, related to Results and Figure 3. The patellar tendon samples (n=3 or 4) of female mice show high similarity of transcriptomic dynamic compared to male mice after puncture-induced injury, where seven cluster of functionally different gene sets can be identified. Gene expression trends within each cluster were analyzed. Top GO terms were determined based on the adjusted p value.

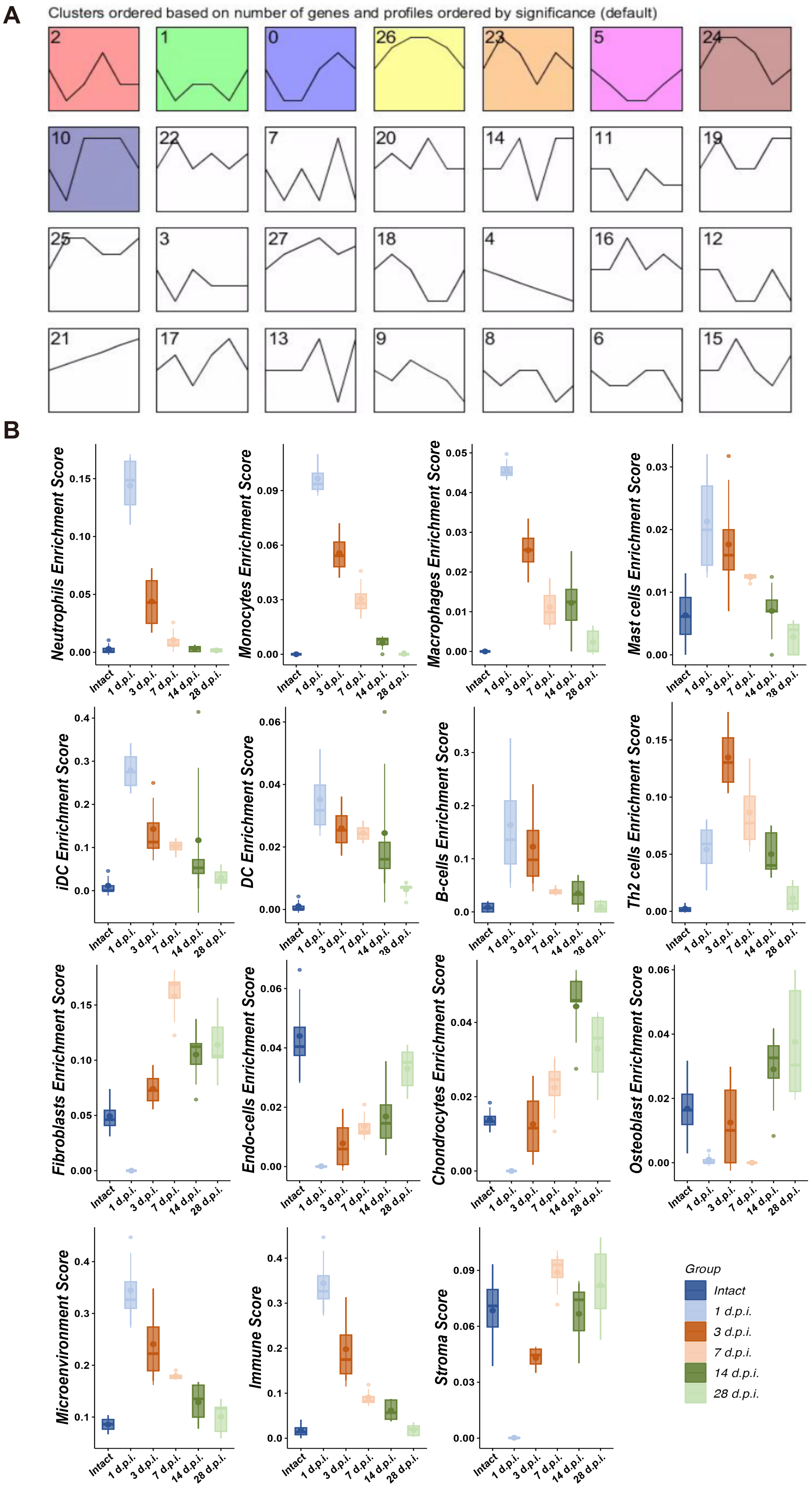
A
Clusters ordered based on number of genes and profiles ordered by significance (default)
2
1
0
26
23
5
24
10
22
7
20
14
11
19
25
3
27
18
4
16
12
21
17
13
9
8
6
15
B
Neutrophils Enrichment Score
Monocytes Enrichment Score
Macrophages Enrichment Score
Mast cells Enrichment Score
iDC Enrichment Score
DC Enrichment Score
B-cells Enrichment Score
Th2 cells Enrichment Score
Fibroblasts Enrichment Score
Endo-cells Enrichment Score
Chondrocytes Enrichment Score
Osteoblast Enrichment Score
Microenvironment Score
Immune Score
Stroma Score
Intact
1 d.p.i.
3 d.p.i.
7 d.p.i.
14 d.p.i.
28 d.p.i.
Group
Intact
1 d.p.i.
3 d.p.i.
7 d.p.i.
14 d.p.i.
28 d.p.i.

Figure S4. STEM-based clustering and xCell result, related to Results and Figure 3.

(A) Results of STEM clustering of differentially expressed genes for pairwise comparisons of different time after injury, profiles with colored background represent statistical differences. Red background corresponds to cluster 2, linked to transcription and translation. Green background corresponds to cluster 1, linked to metabolism. Light purple corresponds to cluster 0, linked to development and vascularization. Yellow background corresponds to cluster 26, linked to cell division and cell cycle. Orange background corresponds to cluster 23, linked to RNA processing. Pink background corresponds to cluster 5, linked to to muscle development and differentiation. Brown background corresponds to cluster 24, linked to stress response, immunity, and inflammation. Dark purple background corresponds to cluster 10, linked to extracellular matrix.

(B) xCell deconvolution for immune infiltration analysis reveal relative immune cell proportion.

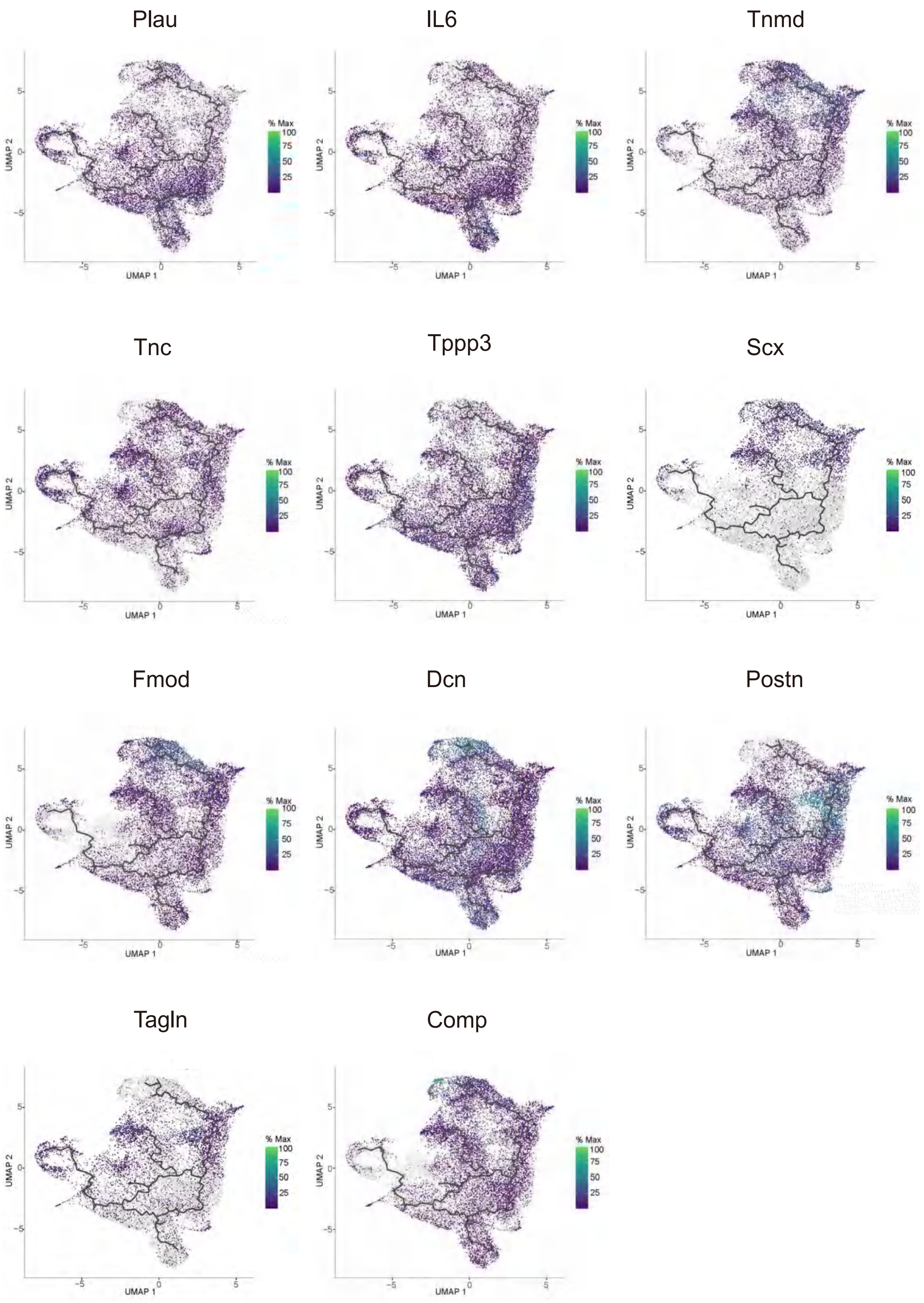

Figure S5. Relative expression of genes of interest, including tendon-related genes on the pseudo-timeline for each cell state, related to Results and Figure 6. Tendon related genes show different distribution patterns.

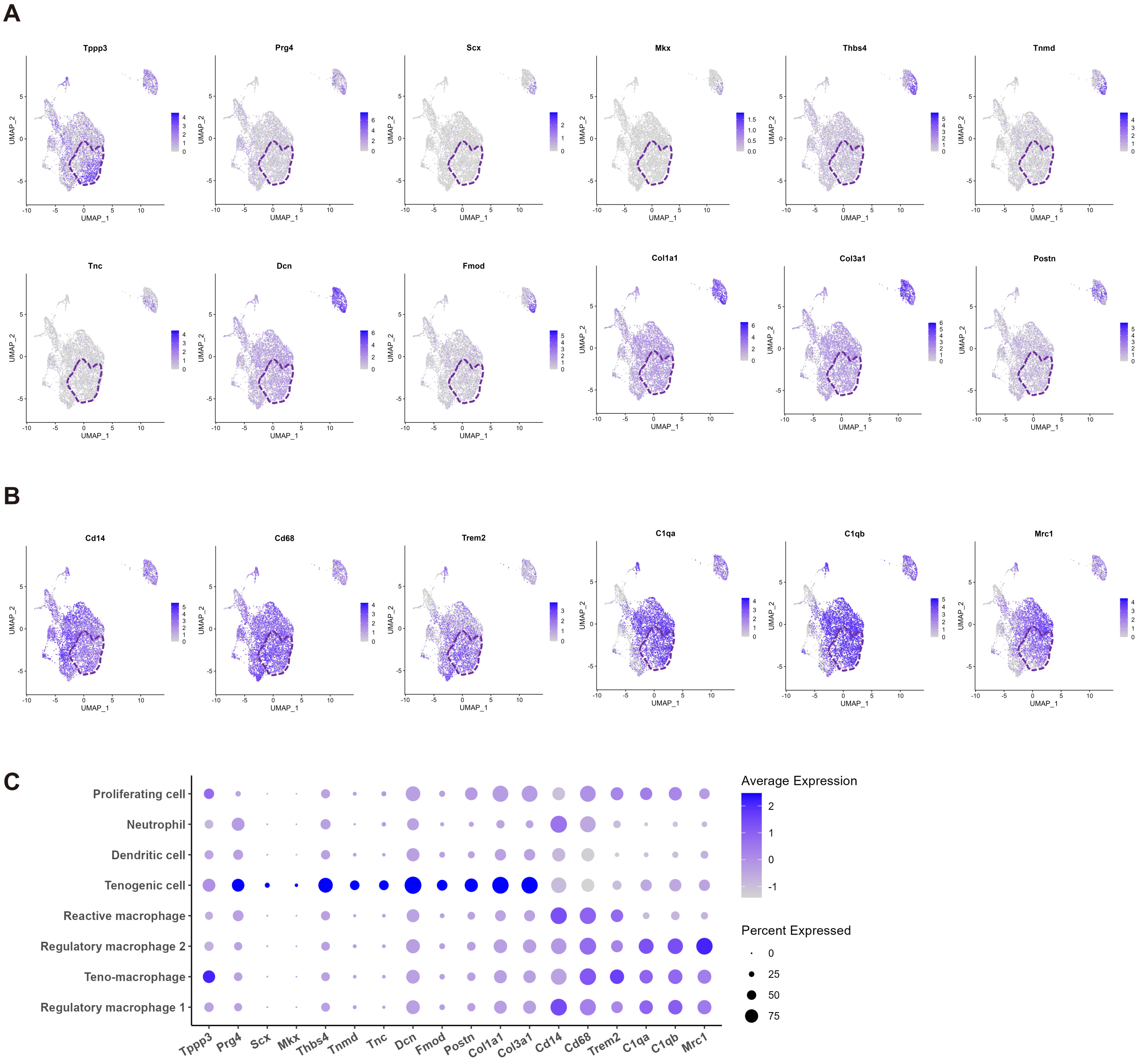

Figure S6. Monocyte/macrophage and tenogenic markers expression level of subsets of myeloid cell population, related to Results and Figure 7.

(A) The macrophage subpopulations show different expression of tendon-related genes, such as Tppp3, Scx, Thbs4. The teno-macrophage subpopulation was circled and exhibit high level of expression of Tppp3 compared to other markers.

(B) The macrophage subpopulations show relatively high expression of macrophage-related genes, such as Cd14, C1qa, C1qb. The teno-macrophage subpopulation was circled and exhibit high level of expression of Cd68, Trem2 and other macrophage markers.

(C) Bubble plot illustrating the expression of tendon or macrophage related marker genes of myeloid population. Darker colors indicate higher expression, and larger circles represent a higher percentage of cells expressing the gene.

Score	0	1	2	3
Fiber structure	Continuous, long fiber	Slight fracture	Moderate fracture	Severe fracture
Arrangement of fibers	Close and parallel	Slightly loose and undulating	Secondary osteoporosis and waved, cross each other	No recognizable features
Degree of nuclear roundness	Long spindle shape	Slightly rounded	Medium rounded	Severely rounded
Degree of inflammation (area of inflammatory cell infiltration)	< 10%	10% - 20%	20% - 30%	> 30%
Blood vessels (angiogenesis infiltration area)	< 10%	10% - 20%	20% - 30%	> 30%
Cell density	Normal	Slight increase	Moderate increase	Severe increase

Table S1. Movin's modified histological scores for tendon, related to Methods Details and Figure 1.

Mesenchymal Cell	Myeloid Cell	Vascular endothelial cell	Pericyte	Lymphatic endothelial cell	Nerve cell
Clec3b	Lyz2	Fabp4	Des	Porx1	Cadm1
Dpt	Cd74	Pecam1	Acta2	Lyve1	Cnp
Pdgfra	Cd68	Cdh5	Itga7	Cd24a	Lgi4
Mki67	Ccl4	Adgrf5	Ttn	Id1	Cpe
Fmod	C1qc	Cav1	Pdgfa	Fgl2	Klf5
Comp	Mrc1	Flt1	Ing2	Cavin2	Plp1
Cilp2	Cd14	Ptprb	cd146	Cldn5	Cryab
Myoc	Fcer1g	Selp	Purb	Lcn2	Cadm4
Tnmd	Arg1	Egfl7	Vcam1	Clca3a1	Lypd2
Prg4	Spp1	Cavin2	Pcm1	Lrg1	Itgb4

Table S2. Representative markers for identifying different cell populationos, related to Results and Figure 4.
